# Advanced FRET normalization allows quantitative analysis of protein interactions including stoichiometries and relative affinities in living cells

**DOI:** 10.1038/s41598-019-44650-0

**Published:** 2019-06-03

**Authors:** Bernhard Hochreiter, Markus Kunze, Bernhard Moser, Johannes A. Schmid

**Affiliations:** 1 Medical University Vienna, Center for Physiology and Pharmacology, Institute for Vascular Biology and Thrombosis Research, Vienna, Austria; 2 Medical University Vienna, Center for Brain Research, Department of Pathobiology of the Nervous System, Vienna, Austria

**Keywords:** Fluorescence imaging, Flow cytometry, Biophysical chemistry, Wide-field fluorescence microscopy

## Abstract

FRET (Fluorescence Resonance Energy Transfer) measurements are commonly applied to proof protein-protein interactions. However, standard methods of live cell FRET microscopy and signal normalization only allow a principle assessment of mutual binding and are unable to deduce quantitative information of the interaction. We present an evaluation and normalization procedure for 3-filter FRET measurements, which reflects the process of complex formation by plotting FRET-saturation curves. The advantage of this approach relative to traditional signal normalizations is demonstrated by mathematical simulations. Thereby, we also identify the contribution of critical parameters such as the total amount of donor and acceptor molecules and their molar ratio. When combined with a fitting procedure, this normalization facilitates the extraction of key properties of protein complexes such as the interaction stoichiometry or the apparent affinity of the binding partners. Finally, the feasibility of our method is verified by investigating three exemplary protein complexes. Altogether, our approach offers a novel method for a quantitative analysis of protein interactions by 3-filter FRET microscopy, as well as flow cytometry. To facilitate the application of this method, we created macros and routines for the programs *ImageJ*, *R* and *MS-Excel*, which we make publicly available.

## Introduction

**Figure 1:**
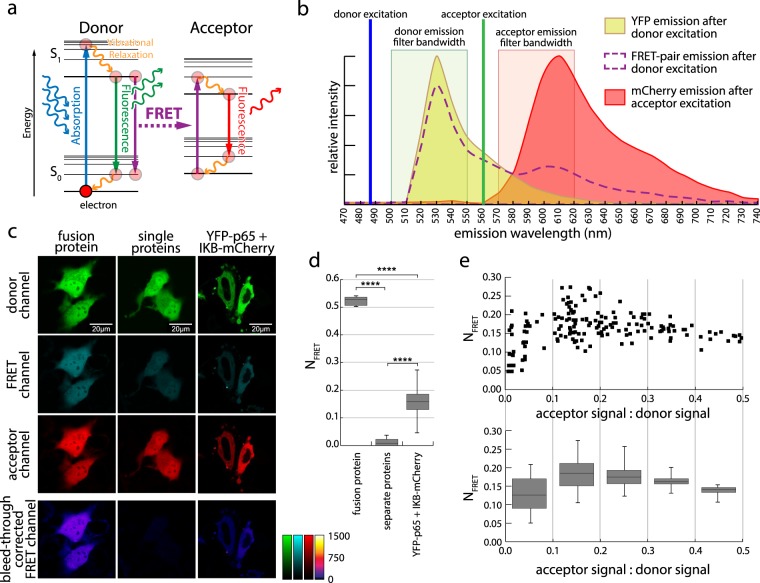
Classical FRET microscopy of protein interactions. (**a**) Jablonski diagram, describing the FRET effect. Excitation of a donor fluorophore raises an electron from the ground state S_0_ to a higher energy state S_1_. Part of that energy is lost by vibrational relaxation. When the electron falls back to S_0_ it can either emit a photon (normal fluorescence) or the energy can be transferred to an electron of a nearby acceptor fluorophore, which is then raised to an excited state S_1_ resulting in fluorescence of the acceptor. (**b**) Emission spectra of donor (yellow) and acceptor (red) alone (after donor or acceptor excitation, respectively), or when acting as FRET pair (dashed line) upon donor excitation. Laser excitation lines, as well as donor and acceptor emission bands of the detector channels are indicated. (**c**) FRET microscopy images of a mCherry-YFP fusion protein (left), YFP and mCherry expressed as non-interacting proteins (middle) and YFP-p65 in combination with its binding partner IκB tagged with mCherry (right). Images of the three detection channels (donor, raw FRET and acceptor) are shown and the calculated corrected FRET^C^ image after subtraction of spectral bleed-through according to Youvan *et al*.^25^. (**d**) Normalized FRET values (N_FRET_ according to Xia *et al*.^27^) for the fusion protein, a negative control of non-interacting proteins and of two interacting proteins (YFP-p65 + IκB-mCherry). Box plots show median values with upper and lower quartiles, error bars represent minimal and maximal values. Statistics: unpaired t-test (****p < 0.0001) (from left to right: n = 43, 54, 184). (**e**) N_FRET_ values for cells presenting with different acceptor to donor ratios of the YFP-p65 + IκB-mCherry FRET pair. Upper panel: Raw data of individual cells as indicated by symbols; lower panel: statistics of N_FRET_ values for the acceptor to donor ratio ranges as indicated by the lines. Box plots are defined as in (**d**) (from left to right: n = 35, 62, 33, 21, 12).

FRET (Förster- or Fluorescence Resonance Energy Transfer) describes a process of radiation-less energy transfer based on dipole-dipole-interactions that can occur from an excited fluorescent molecule (donor) to a suitable acceptor molecule (acceptor)^1^ (Fig. 1a). This process only occurs when the donor fluorescence emission spectrum overlaps with the acceptor excitation spectrum (Fig. 1b) and when donor and acceptor are within close proximity (usually <10 nm). The latter has led to the widespread application of this phenomenon for the identification of molecular interactions^2,3^, as well as the measurement of distances on a molecular scale^4^. While FRET measurements have been applied for the qualitative characterization of many intracellular interactions, the quantification and comparison of these results is particularly challenging due to the complex nature of these processes within living cells and the large number of often unknown variables.

FRET affects the relative intensities of donor and acceptor fluorescence by reducing donor emission and generating acceptor emission upon donor excitation (Fig. 1b and Supplementary Figs 1, 2), and alters the physical properties of the emitted radiation^5,6^. Changes in fluorescence lifetime^7^ or polarization of emitted light^8^ are state of the art ways to determine FRET. However, simple intensity-based methods are still most commonly used, as they are applicable without specialized and expensive equipment while also delivering precise results^9^. One widely used intensity-based FRET method employs the photodestruction of the acceptor molecules (acceptor bleaching) by strong illumination^10,11^. This increases emission of donor fluorescence by eliminating energy dissipation via FRET, but long acquisition times and the destructive nature of the measurement render this method hardly applicable to complex experiments in living cells or set-ups other than microscopy. Furthermore, artefacts and false-positive results can occur due to photoconversion of the acceptor into molecules with donor-like fluorescence properties or by photoactivation of the donor during acceptor bleaching^12,13^.

The most frequently applied technique, named 3-filter FRET (3F-FRET) or sensitized emission FRET^14^, is based on the measurement of three distinct combinations of excitation wavelengths and emission filters: (i) a donor detection channel combining donor-specific excitation with a donor-specific emission filter, (ii) a raw FRET channel employing donor-specific excitation and an acceptor-specific emission filter and (iii) an acceptor channel using an acceptor-specific excitation and an acceptor-specific emission filter (Fig. 1b and Supplementary Figs 1, 2). The experimental design of this method is comparatively easy and, while mostly used in microscopy, can be applied to many different set-ups, including high-throughput methods such as slide-^15,16^ and flow-based cytometry^17–19^. These methods allow the assessment of FRET in entire cell populations, which highly increases the statistical power and permits a more precise extraction of underlying variables from a dataset. A drawback in flow cytometry is the complete loss of spatial information within each single cell, rendering microscopic confirmation of equal distribution a necessary part of such experiments. While specialized equipment also allows the use of fluorescence lifetime measurements in flow cytometry^20,21^, intensity based methods are far more common^22,23^.

The extent of FRET between donor and acceptor is usually described by the FRET efficiency E, which represents the fraction of the total of energy dissipated by the donor that is actually transferred to the acceptor (Eq. 1).

1 $$E=\frac{energy\,transferred\,to\,the\,acceptor}{total\,energy\,released\,by\,the\,donor}$$

General equation for the efficiency of fluorescence resonance energy transfer.

For many experiments it is important to distinguish between the transfer efficiency within a single molecule pair (called FRET efficiency), and the average efficiency of a molecular cohort such as the entirety of fluorescent proteins within a cell or solution, displaying an average of all included single transfers (called apparent FRET efficiency)^11,24^.

3F-FRET does not provide the apparent FRET efficiency as a primary output, due to the differing spectral properties of fluorophores and the use of three independent detection channels. In contrast, methods that depend on single channel measurements, including acceptor photobleaching and fluorescence lifetime microscopy (FLIM), provide E directly^10^. To circumvent this problem, several algorithms were developed, which are considered alternative measures for the FRET efficiency. Widely used normalizations include FRET ^c^ (corrected FRET) by Youvan *et al*.^25^, FRETN by Gordon *et al*.^26^ and N_FRET_ by Xia *et al*.^27^. These corrected FRET measures aim at normalizing the FRET signal to the donor and acceptor signal to overcome variations in expression, but they have been criticized before for not reflecting the actual physical background of FRET^11^. However, due to the ease of application and the implementation of these formulas in most commercial microscopy software and hardware solutions, their use is still widespread^11^.

More advanced normalization procedures developed for the study of molecular interactions calculate FRET efficiencies by normalizing fluorescence intensities with an equipment and fluorophore specific factor called α, which also enables the estimation of molecular ratios of donor and acceptor molecules^28^. There is a wide array of methods to calculate α, most of which require in-depth knowledge about physical parameters of the experimental set-up, including extinction coefficients of the employed fluorophores^15,28–30^. Even with these parameters known, this method is still error-prone^31^, which led to the development of empiric routines that use spectral analysis^32^ or a tandem construct of known FRET efficiency that has been determined by an alternative method such as FLIM or acceptor photobleaching^15,29,30,33,34^. These methods have been thoroughly described and compared in the literature^11,31,35^.

In general, most FRET based experiments can be grouped by their subject of study: (i) single molecule FRET, which measures the transfer between single fluorophore pairs to deduce kinetic properties^36^, (ii) the measurement of FRET in living cells and organisms, employing a wide variety of biosensors to measure biological variables in their physiological environment^37–41^ and (iii) a relatively novel trend of applying FRET to estimate biophysical parameters^42^, such as intra- and inter-molecular distances^4–6,43–48^, stoichiometry of molecular complexes^24,28,32,34,49,50^ or the kinetics and affinities of molecular interactions^51–57^. Many of these biophysical parameters and in particular the binding affinities are mostly measured on extracted proteins outside of their physiological environment. The overlap of live cell measurements and determination of interaction parameters is mostly unexplored with only a few exceptions^29,50,52,57^.

In this work, we demonstrate an easy and standardized method to normalize results obtained from 3-filter based FRET experiments in living cells, show how to obtain additional information by the plotting of a FRET saturation curve, and extract biophysical properties of intermolecular interactions by a fitting algorithm. Furthermore, we explore the capabilities and limitations of commonly applied FRET normalization methods in depth and formulate a mathematical model to demonstrate the influence of various experimental factors. Finally, we also demonstrate the validity of our routine with three distinct biological models of protein interactions.

## Results

### Limitations of commonly used classic FRET-microscopy

In living cells, the investigation of protein-protein interactions by FRET-efficiency measurements is considered a robust method, but with an inherently broad variability of results at single cell level. Standard normalization procedures such as N_FRET_ and FRETN aim at lowering the variance of FRET measurements by relating FRET-values to measures for donor and acceptor concentrations. However, the formation of protein complexes is affected by additional factors, which are not considered by these normalizations. To study these effects in more detail we used the well-described interaction between the transcription factor p65 (RelA) and IκB (inhibitor of κB) from the immunological signaling cascade as model system^58^. Therefore, we transfected HeLa cells with plasmids bearing YFP-p65 and IκB-mCherry and performed classical FRET microscopy.

Correcting the images for spectral bleed-through showed that cells expressing separate YFP and mCherry proteins do not exhibit any remaining FRET^C^ signal, while an mCherry-YFP tandem fusion protein, and the independently expressed interaction partner pair displayed a strong signal (Fig. 1c), demonstrating the suitability of classical FRET microscopy for a qualitative assessment of protein proximity. However, while FRET^C^ provides information on the localization of the interaction, it is not suited for comparison between cells or samples with different expression levels. We applied the N_FRET_ and FRETN normalization routines to our data, which allows the calculation of mean values and statistics (Fig. 1d and Supplementary Fig. 3a). However, when plotted against the ratios of acceptor to donor signal, it became obvious that the classical normalizations fail to provide a plateau of a maximum normalized FRET value at high acceptor concentrations, as would be expected from a saturation of the donor (Fig. 1e). Instead after reaching a peak, the N_FRET_ value declined again with increasing acceptor to donor ratios. FRETN was even more problematic by not showing any clear curve progression and could not detect differences between a fusion protein with strong energy transfer, and a protein pair with a lower transfer (Supplementary Fig. 3b). While some of these problems have been mentioned before^11^, FRETN and N_FRET_ keep coming up in a high percentage of 3-filter based FRET applications. Furthermore, these measures are used in nearly all software solutions provided by microscope manufacturers (Supplementary Table 1). Thus, we can conclude that the classical FRET measures of N_FRET_ and FRETN are not suited for advanced evaluation of FRET experiments with varying acceptor to donor ratios characteristic for most live cell applications. They should only be used for a rough qualitative assessment or studies with FRET-biosensors, which have a constant molecular donor/acceptor ratio.

### Simulation of binding reactions and FRET experiments

**Figure 2:**
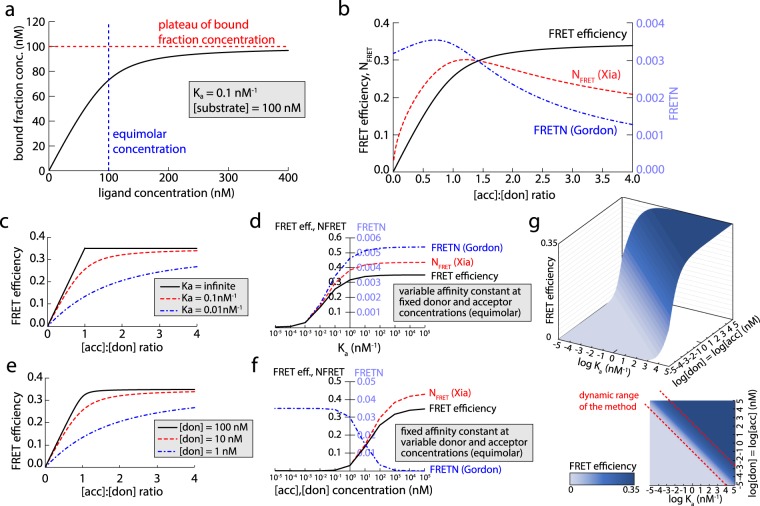
Simulation of the behavior of interacting molecules and the respective FRET values using different normalization routines. (**a**) Formation of a bimolecular complex according to the law of mass action for specified ligand concentrations and affinity. (**b**) Comparison between traditionally normalized FRET efficiency values (N_FRET_ according to Xia^27^ and FRETN according to Gordon^26^) and the theoretical apparent FRET efficiency of a population based on the same parameters across diverse acceptor to donor ratios as calculated by our mathematical simulation. (**c**) Calculated apparent FRET efficiency plotted against acceptor to donor ratios for bimolecular complexes with different affinities as indicated. (**d**) Curve progression of N_FRET_, FRETN and apparent FRET efficiency as a function of the affinity constant at constant, equimolar acceptor and donor concentrations. (**e**) FRET efficiency curves for different constant donor concentrations at varying acceptor to donor ratios ([acc] = 0.01 to 400 × [don]), K_a_ = 1 nM^−1^, FRET_max_ = 0.35. (**f**) Effect of different equimolar concentrations of donor and acceptor on N_FRET_, FRETN and apparent FRET efficiency values. [don] = [acc], K_a_ = 1 nM^−1^, FRET_max_ = 0.35. (**g**) Co-dependence of affinity, reactant concentration and apparent FRET efficiency. Different affinities can only be distinguished where they have a profound impact on FRET efficiency. The sensitivity of FRET measurements is restricted to areas within a dynamic range of affinities (strong slope of the curve), but the positioning of this dynamic range changes with the combined level of donor and acceptor (FRET_max_ set as 0.35).

As traditional FRET measures were unable to reflect the progressive saturation of donor molecules properly, we decided to investigate their behavior and relation to the FRET efficiency further. We generated a mathematical simulation to predict the apparent FRET efficiency and the result of N_FRET_ and FRETN normalization procedures for a population of molecules reflecting an individual cell. Therefore, the concentration of donor and acceptor molecules, the affinity between donor and acceptor and a FRET efficiency for the donor-acceptor pair at complete saturation are defined. First, the simulation uses the law mass action to calculate the fraction of donor molecules engaged in an interaction with an acceptor molecule, which reflects the saturation curve (Fig. 2a). Next, the FRET efficiency of the whole population is predicted by multiplying the maximal FRET efficiency with the fraction of donors engaged in a complex. Finally, the discrete values of different FRET measures are calculated based on the calculated FRET within the population and the known values characterizing this population ([don], [acc], Ka).

The curve progression of FRET efficiency presents equal to a classical binding curve, whereas N_FRET_ and FRETN are distorted due to their way of normalization, displaying the same decline in higher acceptor fractions we experienced in our live cell measurements (Fig. 2b). In contrast, the extrapolated plateau of the apparent FRET efficiency at high acceptor fractions, which we termed FRET_max_, reflects a state of complete donor-saturation by the acceptor.

Furthermore, this simulation enabled us to study the contribution of different experimental conditions on the shape of the FRET-saturation curve by varying the respective parameters. We therefore plotted the FRET curves at different interaction affinities K_a_ and total concentrations (Fig. 2c,e and Supplementary Fig. 4). Reduction of either the affinity or the concentration led to a “flattening” of the curve, which is in line with the law of mass action. We also investigated the influence of these values on the FRET results at equimolar concentration of donor and acceptor (Fig. 2d,f). This demonstrated that apparent FRET measures only reflect changes within a certain dynamic range, as very high or low affinities and concentrations have an increasingly diminished impact on the FRET result. Affinity and binding partner concentrations are both influencing the FRET results interdependently, defining a dynamic range in which differences in interaction strength can be measured by FRET (Fig. 2g).

### Correct calculation of the FRET efficiency in live cell populations

**Figure 3:**
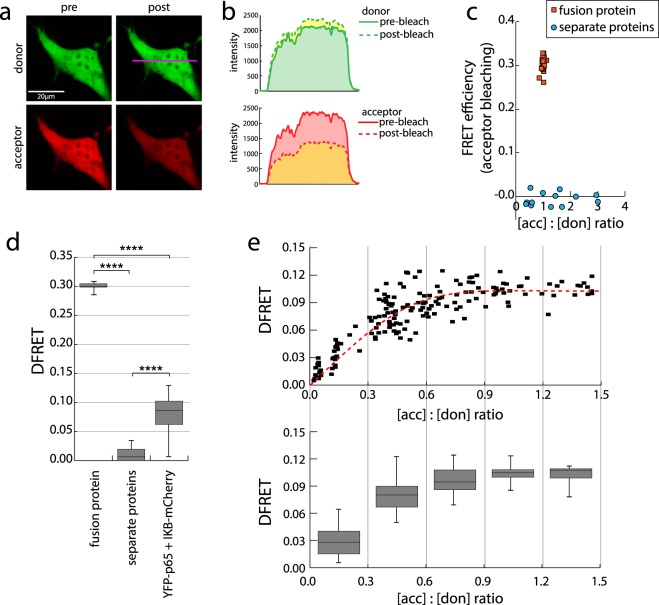
A properly normalized FRET efficiency measure (DFRET) facilitates the depiction of apparent FRET values as saturation curve. (**a**–**c**) Acceptor photobleaching of an mCherry-YFP fusion protein is used as independent method to determine FRET efficiency and the correction factors required for deducing a normalized FRET value (DFRET) from 3-filter FRET measurements. (**a**) HeLa cells were transfected with the mCherry-YFP fusion protein and imaged by laser scanning microscopy before (pre) and after (post) partial photobleaching of the acceptor as described in the Methods section. (**b**) Representative plot of the increase of fluorescence in the donor channel and decrease in the acceptor channel along the profile depicted as pink line in (**a**) after bleaching of the acceptor. (**c**) FRET efficiency calculated from the donor increase after acceptor photobleaching which is plotted against the acceptor to donor ratio for cells expressing the fusion protein (n = 17) or co-expressing YFP and mCherry separate from each other (separate proteins, n = 13). (**d**) Normalized FRET values (DFRET, equivalent to apparent FRET efficiency) obtained by 3-filter FRET microscopy of HeLa cells expressing either the fusion protein, YFP and mCherry separately or the interaction pair YFP-p65 and IκB-mCherry. The values of all cells with different expression levels and ratios are plotted. Box plots show median values with upper and lower quartiles, error bars represent minimal and maximal values. Statistics: unpaired t-test (****p < 0.0001) (from left to right: n = 43, 54, 184).(**e**) Upper panel: Distribution of DFRET values of the same cells over a range of acceptor/donor ratios. Each cell is represented by a symbol. The red line represents the DFRET saturation curve. Lower panel: Box plots for the different ranges of acceptor/donor ratios as indicated by straight lines (from left to right: n = 35, 62, 33, 21, 12). Panel d&e use the same dataset as Fig. 1.

As mentioned earlier, obtaining the apparent FRET efficiency from a population of living cells is not simple in 3-filter FRET experiments due to the fluorophore specifications and the individual properties of the channel detectors. For correct determination of the apparent FRET efficiency and molar ratios between donor and acceptor, the two correctional factors C1 and C2 had to be calculated. The acceptor photobleaching method was applied to cells expressing the mCherry-YFP fusion protein (Fig. 3a,b) to determine an apparent FRET efficiency of 0.30 ± 0.01 while no appreciable FRET was detected in a negative control sample (Fig. 3c). Measuring the same fusion protein in our 3F-FRET experiments enabled us to equate the results, and calculate the factor C1, transforming intensity of the donor channel into an equal measure for the FRET channel, and C2, achieving the same for the acceptor channel.

Using these correction factors in the data previously obtained in our 3-filter FRET experiments for the interaction of YFP-p65 and IκB-mCherry (see Fig. 1), we obtained the apparent FRET efficiencies of each individual cell (termed DFRET to distinguish it from the apparent FRET efficiency determined by other methods), (Fig. 3d). In addition, this method grants information about the real molar ratios of donor and acceptor, enabling us to plot its correlation with DFRET (Fig. 3e). We found that, in contrast to N_FRET_ and FRETN, the DFRET value does not suffer from a decline with increasing acceptor concentrations (Fig. 1e) and perfectly follows the saturation characteristics that are typical for a binding reaction according to the law of mass action.

### FRET measurements by microscopy and flow cytometry

**Figure 4:**
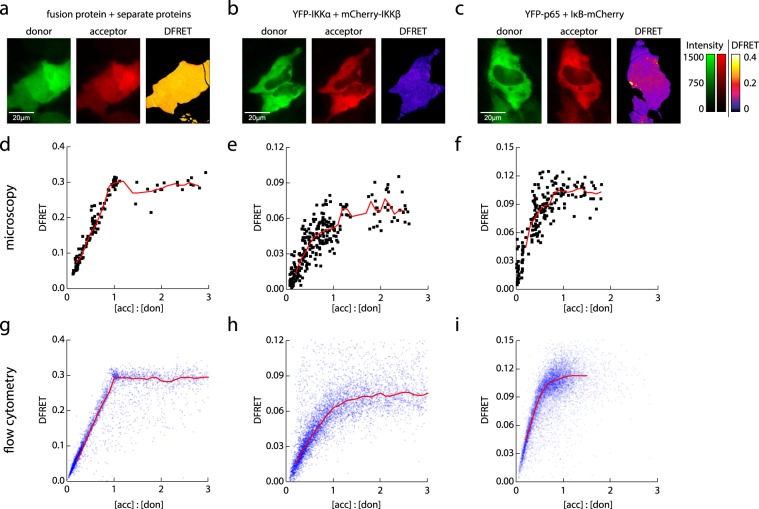
Investigation of three model systems by 3-filter FRET measurements and calculation of DFRET-values confirm the versatility of our approach. (**a**,**d**,**g**) Model of binding partners with infinite affinity: HeLa cells were transfected with expression plasmids to produce the mCherry-YFP fusion protein together with varying amounts of either mCherry or YFP alone. (**b**,**e**,**h**) Binding partners with intermediate affinity interacting in a bimolecular complex: HeLa cells transfected with YFP-tagged IKKα and mCherry-tagged IKKβ as interaction pair. (**c**,**f**,**i**) binding partners with intermediate affinity involved in a trimolecular complex: HeLa cells transfected with the interacting proteins YFP-p65 and IκB-mCherry. (**a**–**c**) 3-filter FRET: fluorescence microscopy pictures depicting the emission in the donor channel (green), the acceptor channel (red) and intensity distribution of DFRET values after multiple correction steps (DFRET: fire LUT 0–0.4). (**d**–**f**) Distribution of DFRET values calculated from FRET microscopy of individual cells over a range of acceptor to donor ratios. Each symbol represents an individual cell. Red line represents moving average. (**g**–**i**) Distribution of DFRET values over different acceptor to donor ratios, which were calculated from intensities obtained by flow cytometry. Relative intensities of donor and acceptor molecules were converted into molar ratios as described in the methods section. Each dot represents an individual cell. Red line represents moving average.

The extraction of quantitative properties of protein-portein interactions from FRET experiments in living cells requires the determination of a correctly normalized FRET value over a broader range of donor/acceptor ratios and a sufficient statistical power, compensating the inhomogeneity of single cells within a population. Since flow cytometry provides fast measurements of thousands of cells, we used this technology in conjunction with our DFRET analysis routine. However, since spatial information is not provided, each sample was also measured on a confocal microscope to ensure co-localization of donor and acceptor throughout the cell (Fig. 4a–c).

To verify the reliability of our normalization routine to study protein-protein interactions, we investigated three distinct types of protein complexes in living cells. The first experimental model mimicked the interaction of two proteins with an infinitely high affinity, upon which one binding partner is completly saturated. To demonstrate this on living cells, we transfected a plasmid encoding our mCherry-YFP fusion construct together with varying amounts of plasmid encoding either YFP or mCherry. The fusion construct cannot dissociate and thereby represents the bound fraction, while the free donor or acceptor molecules represent the free fractions at varying concentration ratios. While this model is not a physiological interaction between two molecules, it mimics one with an infinite affinity (Fig. 4a,d,g). Figure 4a shows representative microscopy images, demonstrating that DFRET represents a normalized FRET efficiency value that is unaffected by local concentration differences. An investigation of DFRET values across the whole cell population reveals an increase with increasing acceptor/donor ratios reaching a sharp inflection point from where on they stay constant (Fig. 4d,g) Thus, the measured values from microscopy and flow cytometry precisely follow the previously predicted behavior at infinite affinity (Fig. 2c).

For the other two models we used real interactions between proteins of the NFκB (nuclear factor kappa B) signaling pathway^58^. To obtain a broad range of donor and acceptor concentrations, we used different plasmid ratios for transfection. First, we investigated the interaction of the two IKK subunits α and β, which are usually associated with each other. However, they also play important roles on their own, suggesting sufficiently low affinities to allow the detection of bound and free populations of both proteins (Fig. 4b,e,h). Compared to the model of infinite affinity, the weaker interaction of IKKα and β is reflected by a smoother transition at the inflection point, governed by affinity and concentration of the two interactors. It is also noteworthy that the FRET efficiency plateau is lower, indicating a larger distance of the fluorophores, which is expected for a larger protein complex compared to a fusion protein with a small linker. The last model presents the data of our earlier experiments (See Figs 1, 3 and Supplementary Fig. 3), investigating the transcription factor p65 (RelA), which appears in homo- or hetero- dimeric complexes, bound to an inhibitory molecule of the IκB family, thereby forming a trimeric complex^58^. This interaction is of interest as it diverts from the 1:1 complex stoichiometry, leading to a curve that is shifted to the left (Fig. 4c,f,i).

The use of flow cytometry provides a much larger dataset, which allows a clearer picture of the saturation behavior of DFRET across a larger range of acceptor to donor ratios (Fig. 4). The data obtained also demonstrates the previously described different curve progressions of N_FRET_ and FRETN respectively (Supplementary Fig. 5). Similar to the predictions from our simulation, N_FRET_ features a peak rather than a saturation plateau, while data depicted as FRETN basically forms a cloud featuring a high spread due to the high impact of variations in expression in each cell (compare Fig. 2 and Supplementary Fig. 4). This again underlines the importance of a dataset that covers a broad range of acceptor to donor ratios, in order to obtain a full picture of the interaction.

### Fitting of FRET values to obtain independent, quantitative measures

**Figure 5:**
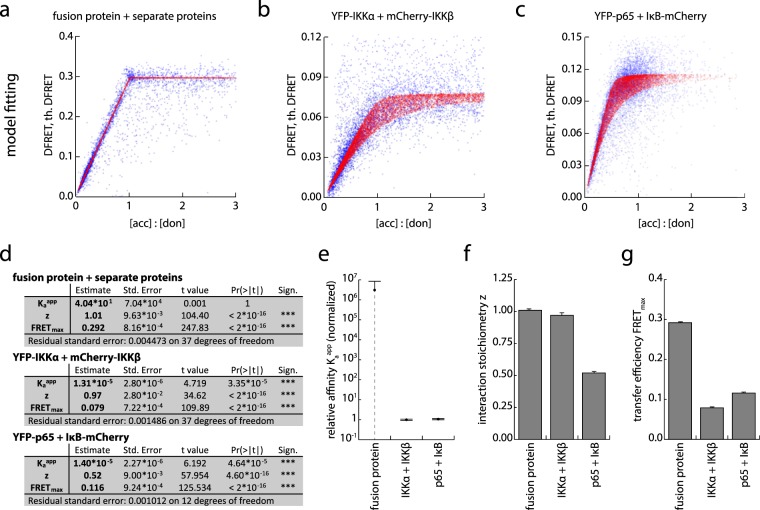
Fitting of properly normalized DFRET values to the mathematical model allows deducing the maximum FRET efficiency, the stoichiometry of the interaction and the apparent affinity. (**a**–**c**) The data-sets depicted in Fig. 4g–i were subjected to retro-fitting against the mathematical model thereby leading to theoretical DFRET values as shown by red dots. The blue dots represent the original data. Residual errors and aggregated curves of real and fitted values are shown in Supplementary Fig. 6. (**d**) Based on the mathematical model, the fitted DFRET values can be used to calculate apparent affinity constants K_a_^app^ (in arbitrary units), as well as a z-factor defining the stoichiometry of the interaction, and a maximum FRET value (FRET_max_) at complete donor saturation providing information on the mean distance between the fluorophore centers. The fitting also provides standard errors of the estimate and statistical parameters associated with tests for significance, including t value (t value) and p value (Pr(>|t|) of the applied t-test. (**e**–**g**) Depiction of the calculated relative affinities K_a_^app^ (**e**), the stoichiometry z (**f**) and the DFRET at total donor saturation (FRET_max_) (**g**) that have been obtained by the fitting of the DFRET data set for the three model systems. Error bars represent std. error of fitted estimates. Error bar for fusion protein is dashed to represent incomplete depiction as it reaches below 0 (not depictable by log scale). The large error of the fusion protein affinity fit results from the dynamic range of the method (Compare Fig. 2). The further away from this range a measured affinity is, the less accurate the prediction becomes, thereby resulting in a huge margin of error for a system with a virtually infinite affinity as described in more detail in the main text.

While a DFRET-saturation curve allows a better depiction of a single molecular interaction, independent physical properties of the interaction cannot be delineated directly, rendering comparison of different samples with varying expression levels impossible. The above described FRET simulation allows the prediction of the DFRET result when biophysical parameters such as the stoichiometry of the interaction (z), the plateau value FRET_max_, and the affinity constant K_a_ of the interaction are known. Contrarily, if the DFRET and concentrations of the interaction partners are known for a reasonably high number of data points, the simulation should be able to predict these basic parameters of the interaction. We devised a routine to retrofit measured intensities and DFRET results into the simulation model, which results in an estimate for these three parameters. The calculated result for the affinity constant K_a_ is a relative value as we are using intensities in the fitting instead of concentrations, which are difficult to obtain in living cells. However, the obtained apparent affinity K_a_^app^ should be linearly correlated to the real affinity constant K_a_, rendering it a useful measure for independent comparison of experiments, as long as they are measured with the same equipment set-up, or are properly normalized. We applied this data fitting method to the results of the three previously described model protein complexes and obtained variables that reflect the difference in these three types of interactions (Fig. 5 and Supplementary Fig. 6). For the interaction stoichiometry z, the model of infinite affinity and the interaction between IKKα and IKKβ fitted close to 1, representing the expected equimolar interaction. The interaction between p65 and IκB fitted close to a z value of 0.5, reflecting the trimeric complex in which two p65 molecules interact with one IκB molecule. The calculated FRET_max_ value, which reflects the average transfer efficiency within a complex, of the model of infinite affinity was 0.2921 ± 0.0008, which is close to the FRET efficiency as determined by acceptor photobleaching (Fig. 5d,f), which is expected due to the use of the fusion protein for normalization. The other two interactions displayed much lower FRET_max_ values of 0.0793 ± 0.0007for the IKKα/IKKβ complex, and 0.1160 ± 0.0009 for the interaction between p65 and IκB. These results fulfill the expectation, that a larger distance between fluorophores causes a lower FRET_max_ value (Fig. 5d,g) Lastly, we obtained a calculated relative affinity for our model of infinite affinity that was six potencies larger than for the other interactions (Fig. 5d,e). However, this value, features a high degree of uncertainty, which can be explained by the fact that differences cannot be measured reliably when the affinity is outside of the methods dynamic range (Fig. 2g). This not only justifies the existence of a numerical value, but also the high variability and uncertain fitting.

Thus, for all three interactions that we tested, the results obtained by 3-filter FRET measurements combined with our mathematical model are in good agreement with available knowledge on the oligomerization status, and the expected shapes of the DFRET saturation curves. These results demonstrate that our novel technique of combining the measurement of DFRET with a mathematical fitting algorithm enables us to reliably predict basic descriptive parameters of a protein-protein interaction in the context of living cells.

## Discussion

In this work, we demonstrate a new normalization and evaluation routine for FRET experiments in living cells, which includes an appropriate determination of the apparent FRET efficiency for individual living cells and the subsequent extraction of underlying physical parameters from the entire cell population, including the complex stoichiometry (z), the transfer efficiency of an interaction pair (FRET_max_) and a relative measure for the binding affinity (K^a^_app_). With this approach, information that is usually not available by standard normalization procedures can be retrieved.

Most relevant for the determination of these variables is the use of a FRET-measure that reflects the underlying mechanism of complex formation. While the FRET efficiency of a single interaction pair is governed by the distance and orientation between the fluorophores, the apparent FRET efficiency of a population of interacting molecules contains information on bound and free fractions and hence, allows the calculation of the underlying parameters of interactions.

We employ the 3-filter method of FRET in combination with flow cytometry, enabling the measurement of large cell populations, which would not be feasible with alternative intensity-based methods such as acceptor photobleaching. Due to the use of three distinct acquisition channels, acquired intensity signals cannot be used to determine the FRET efficiency directly. Traditional normalization techniques such as the frequently applied FRETN^26^ and N_FRET_^27^ aim at overcoming this drawback by including donor and acceptor signals into their calculation, but do not follow the logic of molecular interactions and FRET. Our simulation showed that these classical methods only provide qualitative evidence for a close molecular proximity or interaction, whereas DFRET values can be plotted as saturation curve.

To calculate apparent FRET efficiencies, the intensity in the FRET-channel must be related to the total energy emission of the donor. In our study, we employ a normalization routine that is in line with previous work from Hoppe *et al*.^28^ and Zal *et al*.^35^, who used a donor-acceptor tandem construct to empirically assess a donor normalization factor α. However, in our approach two factors C1 and C2 are used to relate the signal intensity of the FRET-channel with that of the donor channel (C1) and the acceptor channel (C2), respectively, enabling the calculation of apparent FRET efficiencies (DFRET) and relative concentrations of donor and acceptor molecules within each individual cell. This allows a more precise evaluation of results compared to classical experiments, as different parts of the population can be used independently to extract quantitative information. Related, although not similar, separate normalization factors (named γ and ξ) have been described previously^11,48^ to determination molar ratios, but were not used not in conjunction with model based fitting to extract properties of the interaction. Our approach is purely heuristic, achieving a simplified version of previously applied formulas that can be obtained without deep insight into the physical background of FRET and knowledge about specific properties of the fluorescent proteins.

Presuming that the DFRET normalization method actually attributes proper FRET-efficiency values and relative donor and acceptor levels to each cell of a population, we can subject such data sets to a fitting procedure based on the law of mass action. The quality of the fit is highly dependent on the original dataset, requiring a reasonably high amount of data, and a good coverage of the saturation curve. Small datasets, as they would result from classical microscopy, can be fitted, but will suffer from high uncertainties for the resulting estimates. The fitting provides three results: (i) FRET_max_ represents the DFRET at total saturation of donor and corresponds to the extrapolated plateau of the saturation curve. It is mainly governed by the distance and orientation between donor and acceptor. (ii) z defines the molecular ratio of acceptor and donor molecules within the complex and is indicated by the shape of the curve, and mainly its position on the x axis. However, this ratio must not be confused with the actual stoichiometry, as multiples of the molar ratio cannot be recognized. (iii) K_a_^app^ is a relative measure of the interaction strength and correlates to the actual affinity constant K_a_. It correlates with the slope of the saturation curve for comparable total intensities of donor and acceptor, because the curve shape also drastically changes with the latter. Additionally, K_a_^app^ can only be estimated within a certain range, where changes in affinity have a clearly distinguishable effect on the curve shape (see Fig. 2).

Independent FRET-measurement approaches have been suggested for each of these values, but a routine to extract all parameters concomitantly in living cells has not been introduced yet. The FRET efficiency of an interacting donor-acceptor pair, represented by FRET_max_, is a heavily utilized measure in FRET experiments as it relates to the distance between the two fluorophores. The exact assessment of molecular distances is of importance in structural biology, but is accompanied with many problems in regard to the macromolecular sizes of proteins^4,44,46,47^. Even without relating FRET_max_ to the distance, it can be used to illuminate processes of conformational change or as a read-out of FRET-biosensors^37^. A large advantage of the presented method is that the extraction of FRET_max_ from a fit of all available data points allows its extrapolation from an incomplete curve, even when none of the data-points actually reaches the plateau, either due to a low affinity, or the inability to provide acceptor in high enough excess. Complex stoichiometry represents the second important parameter, which is most commonly applied to analyze the oligomerization status of receptors^24,31,45^. While the presented method does not provide specific advantages for the determination of interaction stoichiometry on its own, it provides it in conjunction with other values that provide a better description of the interaction in its entirety.

The last basic parameter is the interaction affinity, as depicted by the affinity constant K_a_ or its reciprocal, the dissociation constant K_d_. The affinity is independent of the concentration of involved reactants and is therefore used as a comparative value of interaction strength. Affinities between proteins have been amply studied by FRET, but a quantification of the interaction strength has rarely been performed in living cells, due to the inability to determine the intracellular concentrations of donor and acceptor proteins properly. Most approaches used isolated proteins^51,55,56,59–61^, thereby not reflecting the physiological environment of living cells, which can lead to a vastly different behavior. Some studies have attempted to measure affinity in live cells, but they all come with certain drawbacks, requiring known concentrations, or at least uniform expression. Chen *et al*.^50^ described a pixel-by-pixel determination of K_d_, but required precise FLIM measurements and worked with fixed and permeabilized cells with added recombinant protein thus not representing a physiological protein interaction. Experiments in bacteria by Lin *et al*.^52^ have used brightness selection to assure equal expression. Butz *et al*.^29^ presented a very advanced method similar to our approach, but employed a Langmuir model to extract quantitative measures, which generally requires one of the interaction partners to be of a fixed concentration, rendering its application very limited and error-prone in a population of living cells with differing expressions. Most recently, de las Heras-Martínez *et al*.^53^ presented an impressive FLIM based method for the determination of K_d_. However, their technique suffers from a long acquisition time and is therefore only applicable in fixed cells or on otherwise immobilized molecules.

Another noteworthy method was previously described by Szalóki *et al*.^57^. They use a similar approach to the one presented here to identify the dimerization dynamics of two transcription factors, including the use of flow cytometry to create FRET titration curves. In contrast to our method, they applied a different mathematical approach and used aggregated curves instead of the totality of the measured cell population to obtain affinities. Without modification, this approach does not allow determination of an unknown complex stoichiometry and the aggregation of data can lead to insecure fitting in a population with diverse expression patterns, which is no problem for our method. On the other hand, they combine their measurements with FCS (Fluorescence correlation spectroscopy) and immunostaining to determine proper concentrations of labeled and unlabeled (endogenous) molecules, which provides an estimate of the real dissociation constant rather than a relative one. When the necessary advanced equipment is available, a combination of both methods could provide a very powerful technique to determine molecular affinities for any type of interaction.

The method presented here allows the concomitant investigation of entire populations of living cells, each presenting with a specific expression level of donor and acceptor proteins. However, the method actually takes advantage of this inherent variability of expression levels, thus converting a traditional weakness of FRET efficiency measurements into a specific strength. Nonetheless, the method is still accompanied by some drawbacks due to the ignorance of actual concentrations. Thus, normalization and fitting are exclusively based on relative levels of donor and acceptor proteins, resulting in a dimensionless apparent affinity. However, in living cells the actual interaction strength is modulated by many factors, such as the cytosolic viscosity, rendering a relative comparison of the affinities of different binding partners or at different timepoints a top priority. Such comparisons can actually be deduced from K_a_^app^, as the unknown factor by which it correlates with K_a_ is constant within the confines of each experimental set-up. However, further improvements on the method and additional normalization might abrogate this limitation in the future.

Overall, our novel method of evaluation, combined with the acquisition of large data sets, either by microscopy or flow cytometry, allows the determination of robust measures of protein-protein interaction far beyond a mere qualitative assessment. This opens the door for many different study-designs that compare different interacting pairs or track their behavior within their natural environment (i.e. living organisms) through various conditions in a way that was not possible previously.

## Methods

### Used Plasmids

All the plasmids that we used are available from the collection of the Schmid lab. A databank containing these is available online under http://www.meduniwien.ac.at/user/johannes.schmid/. Used plasmids and maps are listed in Supplementary Fig. 11.

### Preparation of cell samples

All experiments were done in HeLa cells, clone ACC 57. Cells were passaged one day prior to transfection, reaching a confluence of 70–80% on the day of transfection. For microscopy experiments, cells were seeded in ibidi glass bottom 8-well slides. For flow cytometry, cells were seeded in 24 well multi-well plates. To achieve coverage over a wide range of acceptor to donor ratios, plasmids bearing the donor or acceptor were transfected in five different mass ratios: 5:1, 3:1, 1:1, 1:3 and 1:5. Transfection was achieved with TurboFect™ Transfection Reagent (Thermo Scientific™, Catalog number: R0531) according to product specifications.

### Microscopic image acquisition and evaluation

Microscopy was done on a Nikon A1 R+ laser scanning confocal system equipped with 12-bit detectors using a 60x plan apochromatic oil immersion objective (NA1.4). The donor channel was acquired with excitation at 488 nm and a 525/50 emission filter. The FRET channel was acquired with excitation at 488 nm and a 595/50 emission filter. The acceptor channel was acquired with excitation at 561 nm and a 595/50 emission filter.

Acceptor photobleaching measurements were done on the Nikon A1, using a 60x plan apochromatic oil immersion objective (NA1.4). Photodestruction was applied at a wavelength of 561 nm at 100% laser power for one second (Laser: Melles Griot 85-YCA-020, 20 mW output at 561 nm ± 0.5 nm).

Evaluation of images was done using the *Fiji* software package of *ImageJ* (https://fiji.sc/), and a set of self-written FRET macros that are freely available under GPLv3 (General Public License version 3) on *GitHub* under (https://github.com/BHochreiter). A description and protocol on usage of these macros for evaluation is provided (Supplementary Fig. 7).

### Flow-cytometry acquisition and evaluation

Flow-cytometry based FRET measurements were done on a CYTOFLEX S instrument (Beckman Coulter, Ser. Nr. AW19039, using the following channel setups. Donor channel: 488 nm laser excitation, 525/40 BP emission filter (505–545 nm), FRET channel: 488 nm laser excitation, 610/20 BP emission filter (600–620 nm), acceptor channel: 561 nm laser excitation, 610/20 BP emission filter (600–620 nm).

The *CytExpert* software (https://www.beckman.com/coulter-flow-cytometers/software) was used for data acquisition and gating of analyzed populations. The FlowPy software (http://flowpy.wikidot.com/) was used for extraction of data from FCS file format into tab delimited txt format.

### Acceptor bleaching evaluation

As an alternative method to determine the FRET efficiency, we used the acceptor photobleaching method, which utilizes the direct comparison of the emission intensity of the donor before and after photodestruction of the acceptor fluorophore, whereupon most measurement and instrument caused distortions are irrelevant and therefore the result is a direct correlate of the physical process. However, many fluorophores exhibit certain spectral abnormalities when illuminated with a strong light source, which have to be accounted for before FRET analysis. Donor fluorophores can sometimes be co-bleached, or on the contrary be photoactivated by illumination with acceptor specific wavelength, leading to a change in fluorescence signal without the presence of FRET. Another phenomenon is photoswitching of the acceptor after bleaching, where, instead of losing its emission, the acceptor shifts to another emission profile that can often be detected in the donor channel^12,13^.

The correctional factors df (co-bleaching and photoactivation of donor) and af (photoswitching of acceptor) are used to account for these effects and are determined with samples containing donor or acceptor alone.

2 $$df=\frac{{D}_{dpost}-{D}_{dpre}}{{D}_{dpre}}$$

3 $$af=\frac{{D}_{apost}-{D}_{apre}}{{A}_{apre}-{A}_{apost}}$$

where “post” means intensity after bleaching with the acceptor-specific excitation and “pre” the value before.

Improper determination and normalization to these effects can lead to an overestimation of donor increase and hence FRET. Another factor that plays an important role is the fact that in most experiments, not 100% of the acceptor fluorescence can be eliminated by bleaching. Equation 30 normalizes the increase in donor intensity after photobleaching to all three of these factors.

4 $${\rm{\Delta }}{D}_{da}^{c}=\frac{({D}_{da\,post}^{c}-df\ast {D}_{da\,pre}^{c}-af\ast ({A}_{da\,pre}^{c}-{A}_{da\,post}^{c}))-{D}_{da\,pre}^{c}}{1-\frac{{A}_{da\,post}^{c}}{{A}_{da\,pre}^{c}}}$$

${\rm{\Delta }}{D}_{da}^{c}$ represents the amount of donor fluorescence that is lost due to FRET, and can therefore directly be used for the determination of E.

5 $$E=\frac{{\rm{\Delta }}{D}_{da}^{c}}{{D}_{da\,pre}^{c}+{\rm{\Delta }}{D}_{da}^{c}}$$

### Calculation of normalization factors C1 and C2

Donor, FRET and acceptor signals require normalization in order to transform them into the same dimension and enable calculation of relative donor and acceptor concentrations and ratios. C1 and C2 normalize donor or acceptor respectively to the signal in the FRET channel. They require information from a construct of known transfer efficiency and stoichiometry. Each experiment measured included a population transfected with an mCherry-YFP tandem fusion construct which fulfils these requirements.

C1, the donor signal correction factor is calculated from the known efficiency of the construct:

6 $$C1=\frac{{F}^{c}-E\ast {F}^{c}}{E\ast {D}_{da}^{c}}$$

In contrast, C2 is calculated from the inferred knowledge of a fixed 1:1 stoichiometry of the fusion protein

7 $$C2=\frac{{D}_{da}^{c}\ast C1+{F}^{c}}{{A}_{da}^{c}}$$

C1 and C2 are simple multiplicative factors that allow calculation of a molecular ratio between acceptor and donor but are equally used as relative measures of the concentration in the later applied model fitting.

8 $$\frac{[acc]}{[don]}=\frac{{D}_{da}^{c}\ast C1+{F}^{c}}{{A}_{da}^{c}\ast C2}$$

### Calculation of different FRET measures from the acquired signals

Due to the spectral properties of fluorophores, the signals of donor, acceptor and FRET channel have to be corrected for spectral crosstalk (or bleed-through) from the other fluorophore. First, four spectral bleed-through factors are determined using samples containing only donor or acceptor fluorophore. Different nomenclatures for these factors are found in the literature – this work uses S1–S4^31^. S1 and S3 describe the spectral bleed of the donor fluorescence into the FRET and acceptor channel respectively, while S2 and S4 describe the spectral bleed of acceptor fluorescence into the FRET and donor channel respectively. (A list of mathematical parameters in the equations is provided in Table 1)

9 $${\rm{donor}}\,{\rm{into}}\,{\rm{FRET}}\,{\rm{channel}}:{S}_{1}=\frac{{F}_{d}}{{D}_{d}}$$

10 $${\rm{acceptor}}\,{\rm{into}}\,{\rm{FRET}}\,{\rm{channel}}:{S}_{2}=\frac{{F}_{a}}{{A}_{a}}$$

11 $${\rm{donor}}\,{\rm{into}}\,{\rm{acceptor}}\,{\rm{channel}}:{S}_{3}=\frac{{A}_{d}}{{D}_{d}}$$

12 $${\rm{acceptor}}\,{\rm{into}}\,{\rm{donor}}\,{\rm{channel}}:{S}_{4}=\frac{{D}_{a}}{{A}_{a}}$$

**Table 1:** List of variables.

variable	name	description
**physical variables**		
[*don*]	donor concentration	
[*acc*]	acceptor concentration	
*D*_*d*_, *D*_*a*_, *D*_*da*_	donor channel signal	background-corrected signal in the donor channel for sample that contains donor, acceptor or both, respectively
*F*_*d*_, *F*_*a*_, *F*_*da*_	FRET channel signal	background-corrected signal in the FRET channel for sample that contains donor, acceptor or both, respectively
*A*_*d*_, *A*_*a*_, *A*_*da*_	Acceptor channel signal	background-corrected signal in the acceptor channel for sample that contains donor, acceptor or both, respectively
**spectral bleed-through correction**		
*S*_1_	bleed-through factor 1	describes spectral bleed-through from donor into FRET channel
*S*_2_	bleed-through factor 2	describes spectral bleed-through from donor into acceptor channel
*S*_3_	bleed-through factor 3	describes spectral bleed-through from acceptor into FRET channel
*S*_4_	bleed-through factor 4	describes spectral bleed-through from acceptor into donor channel
*D* ^*c*^_*da*_	corrected donor signal	signal in the donor channel, corrected for spectral bleed-through
*A* ^*c*^_*da*_	corrected acceptor signal	signal in the acceptor channel, corrected for spectral bleed-through
*FRET*^*c*^, *F*^*c*^	corrected FRET signal	FRET value corrected for spectral bleed-through according to Youvan *et al*.^25^
**normalized FRET measures**		
*E*	FRET efficiency	
*FRETN*	normalized FRET	normalized FRET measure according to Gordon *et al*.^26^
*N*_*FRET*_	normalized FRET	normalized FRET measure according to Xia *et al*.^27^
*DFRET*	normalized FRET	FRET efficiency calculated via normalization of 3-filter FRET based intensities
**correction factors**		
*G*, α	microscope correction factor	
C1	correction factor 1	correction factor for donor related deviations in 3-filter FRET based experiments
C2	correction factor 2	correction factor for acceptor related deviations in 3-filter FRET based experiments
**Acceptor bleaching measures**		
*df*	donor bleaching factor	factor for the normalization to donor-related deviations in acceptor bleaching based FRET experiments
*af*	acceptor bleaching factor	factor for the normalization to acceptor-related deviations in acceptor bleaching based FRET experiments
Δ*D*^*c*^_*da*_	corrected donor difference	difference in donor intensity after acceptor photobleaching, normalized for donor and acceptor related deviations, and incomplete acceptor bleaching
**model variables and read-outs**		
*K*_*a*_	affinity constant	affinity constant of an interaction, given in M^−1^
${K}_{a}^{app}$	apparent affinity constant	relative affinity constant of an interaction, given in arbitrary units (A.U.)
z	stoichiometry factor	dimensionless factor, describing stoichiometry of acceptor and donor molecules within the complex
*FRET*_*max*_	maximal FRET	Apparent FRET efficiency at complete donor saturation

Donor and acceptor signals within a mixed sample can be corrected for spectral bleed-through by applying these factors.

13 $${D}_{da}^{c}=\frac{{D}_{da}-{S}_{4}\ast {A}_{da}}{1-{S}_{3}\ast {S}_{4}}$$

14 $${A}_{da}^{c}=\frac{{A}_{da}-{S}_{3}\ast {D}_{da}}{1-{S}_{3}\ast {S}_{4}}$$

If a donor does not give any signal in the acceptor channel and vice versa, meaning that S3 and S4 are 0, then this step can be omitted, and the signals can be used directly after background correction.

The corrected donor and acceptor signals can be used to separate the actual FRET signal from spectral bleed-through in the FRET channel, according to Youvan *et al*.^25^.

15 $$FRE{T}^{c}={F}_{da}-{D}_{da}^{c}\ast {S}_{1}-{A}_{da}^{c}\ast {S}_{2}$$

The normalized FRET measure depicted as FRETN by Gordon *et al*.^26^ normalizes FRET^c^ to the product of donor and acceptor signal and introduces the G factor (also named α), which normalizes the signals to the FRET signal to account for measurement- and instrument-dependent deviations. It is mostly given in the form below (eq. 16).

16 $$FRETN=\frac{FRE{T}^{c}}{G\ast {D}_{da}^{c}\ast {A}_{da}^{c}}$$

N_FRET_ by Xia *et al*.^27^ relates FRET^c^ to the square root of the product of donor and acceptor signal, but does not include a normalization factor such as G, distorting the results and rendering the comparison of values from different experiments as well as the fit to a model algorithm very difficult.

17 $${N}_{FRET}=\frac{{F}^{c}}{\sqrt{{D}_{da}^{c}\ast {A}_{da}^{c}}}$$

The calculation of the FRET efficiency from the intensities of the 3-filter based method is called DFRET in this work, to distinguish it from the FRET efficiency measured by alternative methods and as it is based on the corrected donor fluorescence (in presence of acceptor).

18 $$DFRET=\frac{{F}^{c}}{C1\ast {D}_{da}^{c}+{F}^{c}}$$

### Simulation of FRET experiments

For the simulation of FRET experiments, we generated a mathematical model based on the generalized form of the law of mass action, which describes the state of all chemical interactions at equilibrium.

19 $$A+B\rightleftharpoons \text{AB},\,{K}_{a}=\frac{[AB]}{[A]\ast [B]}$$

The state of the equilibrium, or more precisely, the concentrations of free and bound fractions of molecules is dependent on the concentrations of reactants, as well as their affinity, described by the affinity constant K_a_ (or its inverse, the dissociation constant K_d_). K_a_ is a constant value for a reaction at a specified temperature and pressure. For the calculation of FRET, we can use our donor and acceptor as A and B respectively.

20 $${K}_{a}=\frac{[complex]}{{[don]}_{free}\ast {[acc]}_{free}}$$

21 $$[complex]=[don]-{[don]}_{free}=[acc]-{[acc]}_{free}$$

When these formulas are combined and solved for [complex], the amount of bound and free species can be determined under a given total concentration of donor, acceptor and a K_a_ value.

22 $${K}_{a}=\frac{[complex]}{([don]-[complex])\ast ([acc]-[complex])}$$

23 $$[complex]=\frac{-\sqrt{{(-[don]{K}_{a}-[acc]{K}_{a}-1)}^{2}-4[don][acc]{K}_{a}^{2}}+[don]{K}_{a}+[acc]{K}_{a}+1}{2{K}_{a}}$$

For the calculation of reactant stoichiometries that are different than 1:1, we added an additional factor z, which describes the amount of acceptor molecules per donor molecule in the complex. Therefore, every [acc] in the formula requires division by z.

24 $$[complex]=\frac{-\sqrt{{(-[don]{K}_{a}-\frac{[acc]}{z}{K}_{a}-1)}^{2}-4[don]\frac{[acc]}{z}{K}_{a}^{2}}+[don]{K}_{a}+\frac{[acc]}{z}{K}_{a}+1}{2{K}_{a}}$$

It is important to note that this way of introducing a stoichiometry factor implies that the multiple donor or acceptor molecules that appear in the complex are already bound to each other before the interaction between donor and acceptor molecules. This formula is not able to depict the affinity of donor or acceptor sub-complexes separately.

25 $$A+zB\mathop{\leftrightarrow }\limits^{unknown}A+{B}_{z}\mathop{\leftrightarrow }\limits^{{K}_{a}}A{B}_{z},\,{K}_{a}=\frac{[A{B}_{z}]}{[A]\ast [{B}_{z}]}$$

Theoretical ${D}_{da}^{c}$, ${A}_{da}^{c}$ and *F*^*c*^ signals can be calculated by applying a fixed value for the maximal FRET efficiency FRET_max_, which represents the FRET efficiency if all donor and acceptor molecules were engaged in a donor-acceptor-complex, as well as the spectral bleed-through factors S1, S2, S3 and S4.

26 $${D}_{da}={[don]}_{free}+[complex]\ast (1-FRE{T}_{max})+({[acc]}_{free}+[complex])\ast {S}_{4}$$

27 $${A}_{da}={[acc]}_{free}+[complex]+(\,{[don]}_{free}+[complex]\ast (1-FRE{T}_{max}))\ast {S}_{3}$$

28 $${F}_{da}=[complex]\ast FRE{T}_{max}+S1\ast {D}_{da}+S2\ast {A}_{da}$$

For the computational simulation, the model can be simplified by setting S1, S2, S3 and S4 to 0, which doesn’t change the mathematical modelling.

From the obtained values, FRET measures were calculated according to Equations 16 to 18.

### Fitting of FRET results

In order to obtain the three quantitative variables K_a_^app^, z and FRET_max_, we retrofitted the results of our measurements into the simulation model. In order to directly use the intensities of donor and acceptor channel, as well as the resulting DFRET for fitting, we slightly modified the formula to obtain the FRET measure instead of the complex concentration.

29 $$don={D}_{da}^{c}\ast C1+Fc$$

30 $$acc={A}_{da}^{c}\ast C2$$

31 $$DFRET=\frac{-\sqrt{{(-don{K}_{a}^{app}-\frac{acc}{z}{K}_{a}^{app}-1)}^{2}-4\,don\,\frac{acc}{z}\,{K}_{a}^{app2}}+don\,{K}_{a}^{app}+\,\frac{acc}{z}\,{K}_{a}^{app}+1}{2{K}_{a}^{app}}\ast \frac{FRE{T}_{max}}{don}$$

Fitting was done via a non-linear least square model, minimizing the deviation of theoretical and real values over several iterations. The model fit can be directly applied to the measured values but should be restricted to a meaningful region around the stoichiometry of the complex. For a simple 1:1 interaction, we apply the model on all values with an acceptor to donor ratio between 0.2 and 2.

### Statistical information

Statistical values for model fit were determined via the non-linear least square fitting algorithm of the software R. Statistical information (where applicable) and n numbers are given in the respective figures or figure legends.

### Implemented software and code

All of the described calculations and evaluations can be done in freely available (*R, ImageJ, CytExpert, FlowPy*) or common (Microsoft Excel) software packages.

FRET calculations, the evaluation of FRET, and the simulations were done in Microsoft Excel (Version 2013). Example datasheets (Supplementary Template 1 & 2) including explanations on use and a sample data set are provided (Supplementary Figs 8–10).

Model Fitting was done in R (https://www.r-project.org/), using the nonlinear least square fit commands. The code used for fitting is provided (Supplementary Note 2).

All written code that was used in this work, including fully automated ImageJ macros and R code sequences are supplied in the supplementary, or available on Github under https://github.com/BHochreiter.

## Supplementary information

Supplementary Information

Dataset 1

Dataset 2

Dataset 3

## Data Availability

The data that support the findings of this study are available from the corresponding author upon reasonable request.
