# Supplementary material for: Advanced FRET normalization allows quantitative analysis of protein interactions including stoichiometries and relative affinities in living cells
Source: Sci Rep. 2019 Jun 3;9:8233. doi: 10.1038/s41598-019-44650-0 (PMC6547726; doi:10.1038/s41598-019-44650-0)
Supplement: Supplementary file 1 — Supplementary Information [file 41598_2019_44650_MOESM1_ESM.pdf]

Supplementary Information

Advanced FRET normalization allows quantitative analysis of protein interactions including stoichiometries and relative affinities in living cells

Bernhard Hochreiter[1], Markus Kunze[2], Bernhard Moser[1], Johannes A. Schmid[1*]

*Corresponding author: e-mail: johannes.schmid@meduniwien.ac.at

[1] Medical University Vienna, Center for Physiology and Pharmacology, Institute for Vascular Biology and Thrombosis Research, [2] Medical University Vienna, Center for Brain Research, Department of Pathobiology of the Nervous System

Contents

Supplementary Table 1 2
Supplementary Figure 1. 3
Supplementary Figure 2. 4
Supplementary Figure 3. 5
Supplementary Figure 4. 6
Supplementary Figure 5. 7
Supplementary Figure 6. 8
Supplementary Figure 7. 9
Supplementary Figure 8. 10
Supplementary Figure 9. 11
Supplementary Figure 10. 12
Supplementary Figure 11. 13
Supplementary Note 1. Fiji Macro for microscopy evaluation 14
Supplementary Note 2. R code for curve fitting of FRET results 16

Supplementary Table 1.
List of FRET normalisations used by commercially available microscopy software packages

Company	Software Name	Normalisation Methods
Zeiss	AxioVision	$FRET^{C}$(Youvan), FRETN (Gordon),N_{FRET} (Xia)
	Zen software	$FRET^{C}$(Youvan), FRETN (Gordon),N_{FRET} (Xia)
Olympus	Fluoview	Acceptor photobleaching
Nikon	NIS Elements	$FRET^{C}$(Youvan), corrected FRET
Leica	Leica Application Suite Advanced Fluorescence	Acceptor bleaching, sensitized emission
Visitron	Visiview	Online Ratio
Picoquant		FLIM, $FRET^{C}$(Youvan),
Metamorph		$FRET^{C}$(Youvan), $FRET^{C}$(Youvan)/Donor

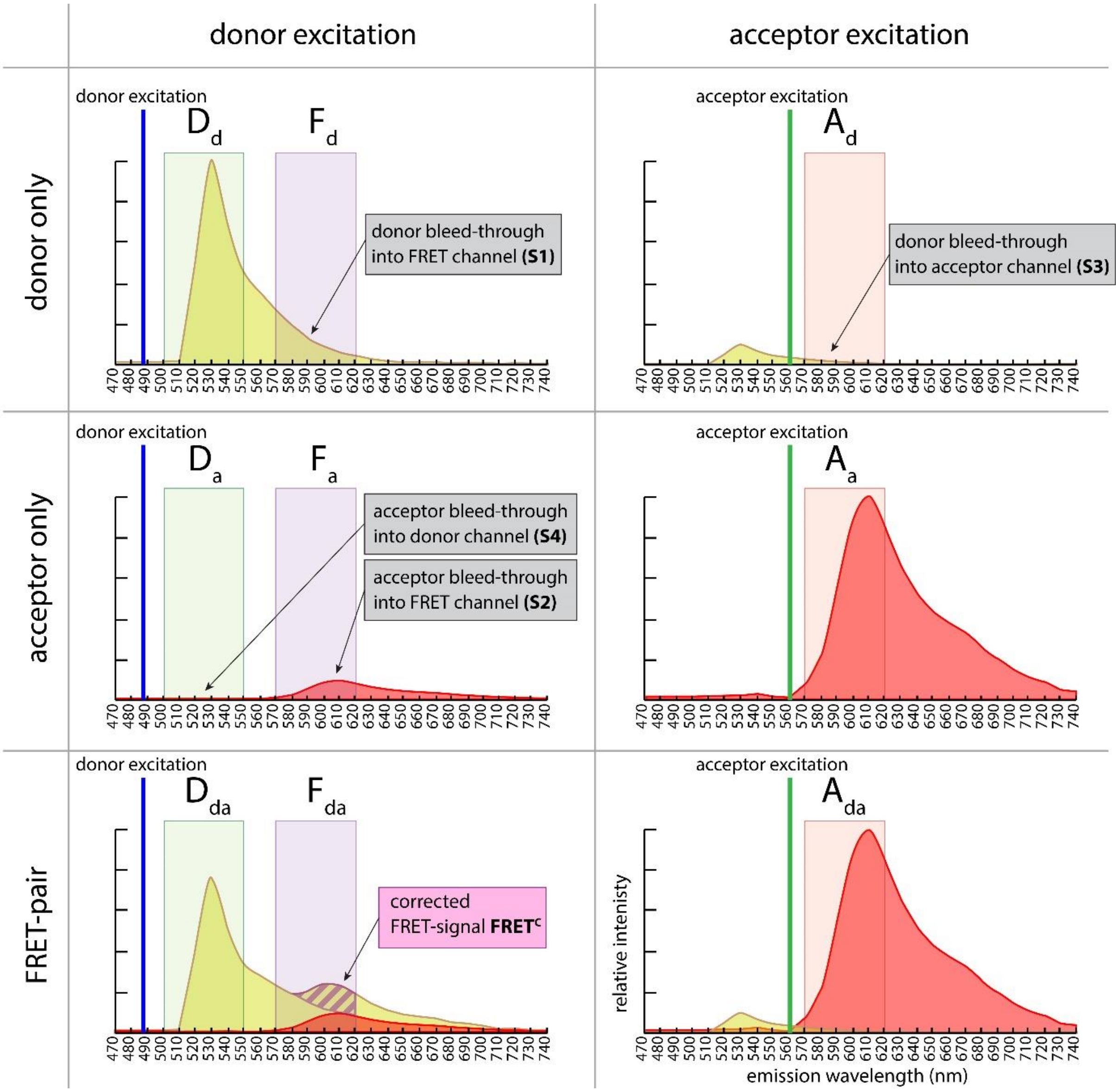

Supplementary Figure 1.

Graphical representation of different signals acquired during 3-filter based FRET experiments and their nomenclature**.** Emission spectra of donor and acceptor fluorophore in the three essential samples: containing only donor (top row), only acceptor (middle row) and the FRET-pair (bottom row). The capital letter describes the detection channel: D = donor channel, F = FRET channel, A = Acceptor channel. The small subscript letter describes the sample content: d = Sample containing only donor fluorophore, a = Sample containing only acceptor fluorophore, da = Sample containing both, donor and acceptor fluorophore.

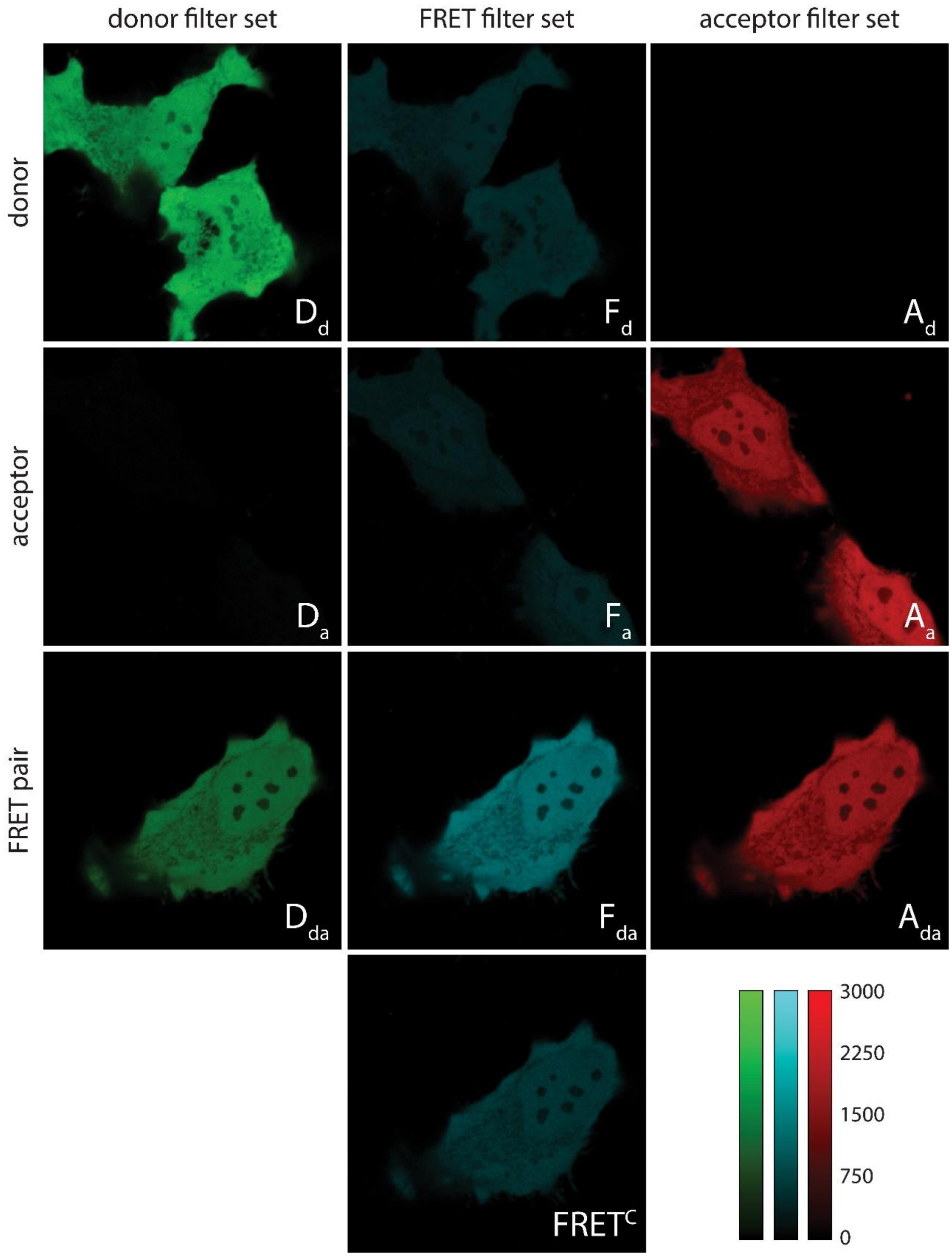

Supplementary Figure 2.

Example images of 3-Filter FRET microscopy for the different detection channels with the nomenclature used in the manuscript. The bottom row represents the bleed-through corrected FRET image without normalization according to Youvan et al. [25]

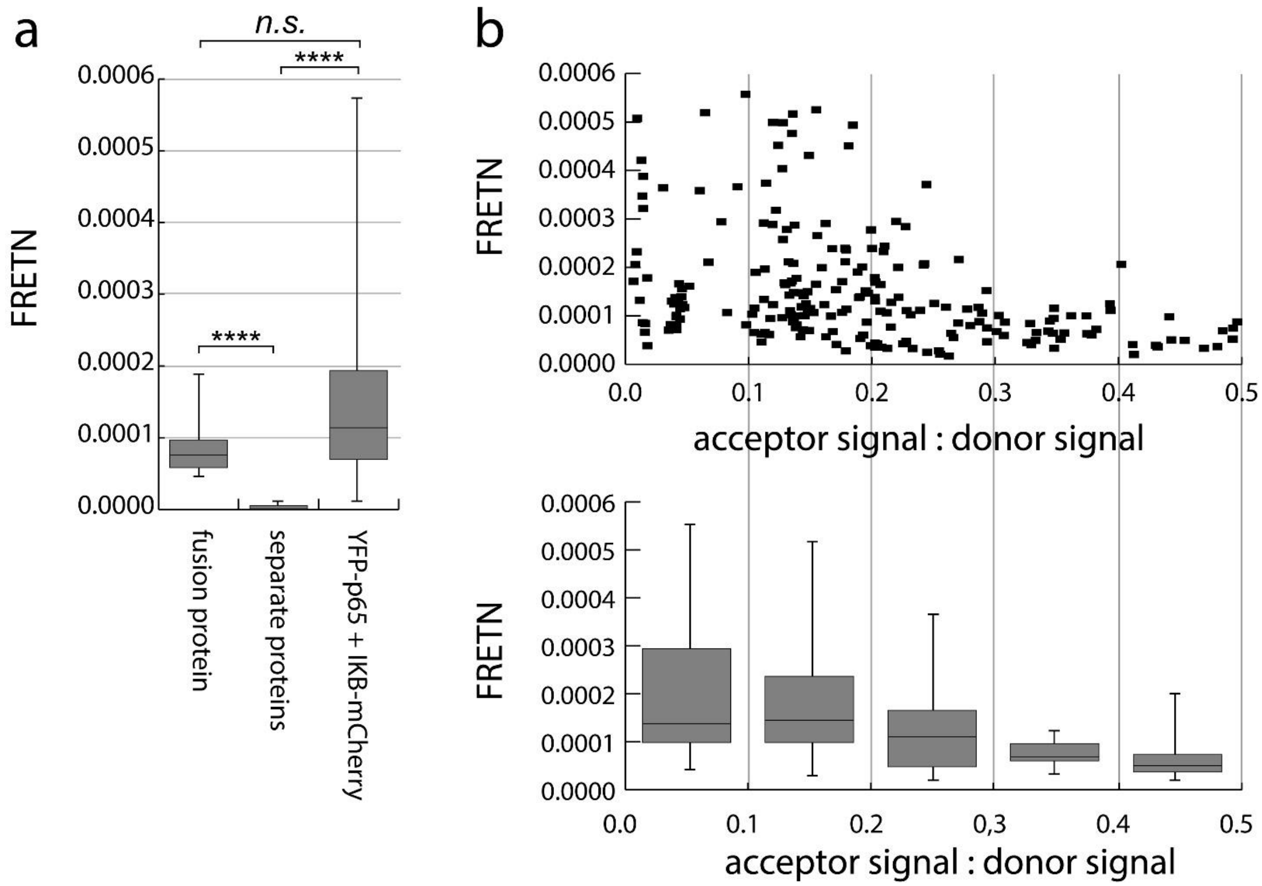

Supplementary Figure 3.
Normalization of the 3-filter FRET microscopy data seen in Fig. 1 according to Gordon et al. [26] (a) Normalized, corrected FRET values (FRETN according to Gordon et al.,) for the fusion protein, a negative control of non-interacting proteins and a FRET of interacting proteins (YFP-p65 + IKB-mCherry). Box plots show median values with upper and lower quartiles, error bars represent minimal and maximal values. (fLTR n=43, 54, 184). (b) FRETN values for different acceptor to donor ratios of the YFP-p65 + IKB-mCherry FRET pair. Upper panel: Raw data of individual cells as indicated by symbols; lower panel: statistics of NFRET values for the acceptor : donor ratio ranges as indicated by the lines. Box plots are defined as in (A) (from left to right n=35,62,33,21,12)

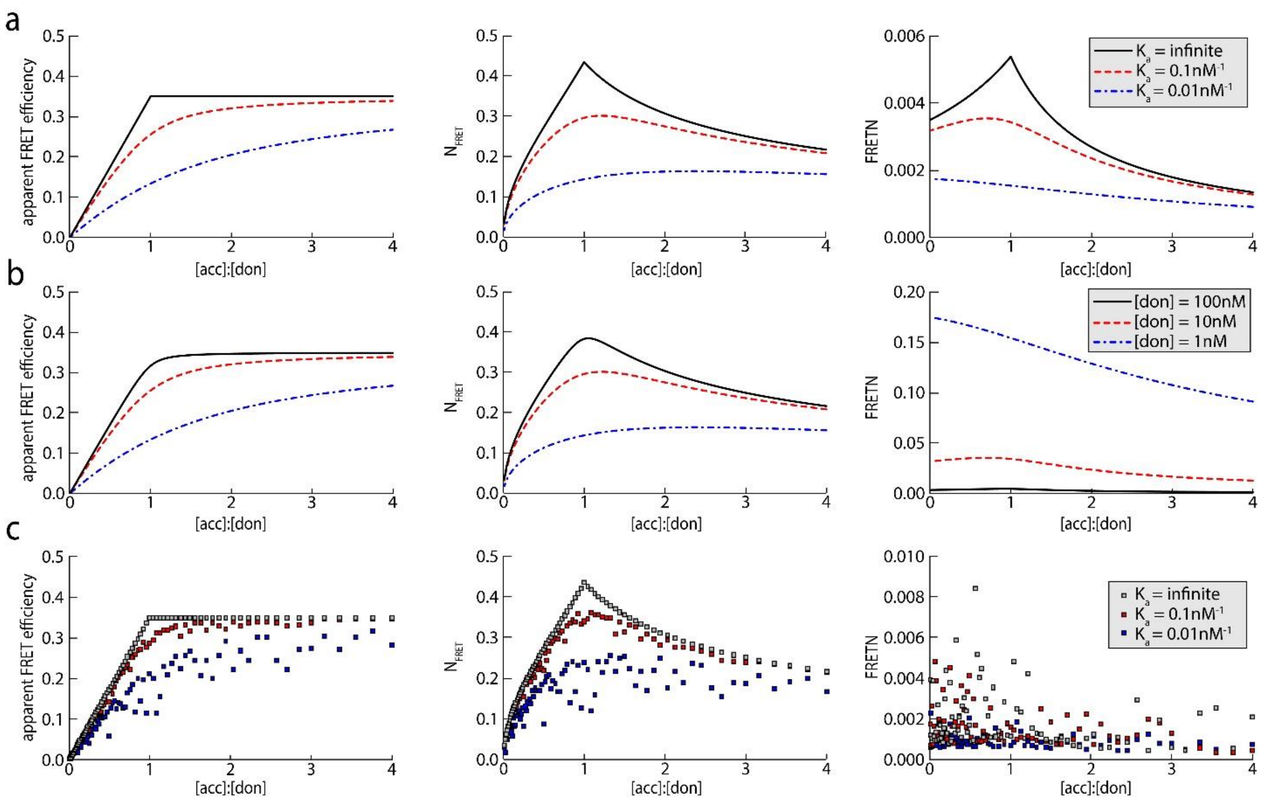

Supplementary Figure 4.

Details on the simulation of different FRET measures. From left to right, plots depict apparent FRET efficiency, N_{FRET} and FRETN plotted against the [acc] to [don] ratio. (a) Effect of different affinities, [Don] = 100nM, K_a=infinite (black line), $0.1nM^{-1}$(red dashed line), $0.01nM^{-1}$(blue dash-dotted line), [acc] is ranging in concentration from 1 to 400 nM, $FRET_{max}$=0.35. (b) Effect of varying donor concentrations, [Don] = 100nM (black line), 10nM (red dashed line), 1nM (blue dash-dotted line), K_a=$1nM^{-1}$, [acc] is ranging in concentration from 0.01 to 400 nM, $FRET_{max}$=0.35. (c) Effect of varying concentration levels on experimental outcome. [don] = ranging randomly from 50 to 500nM, K_a=infinite (grey points), $0.1nM^{-1}$(red points), $0.01nM^{-1}$(blue points), [acc] is ranging in concentration from 1 to 2000 nM, $FRET_{max}$=0.35.

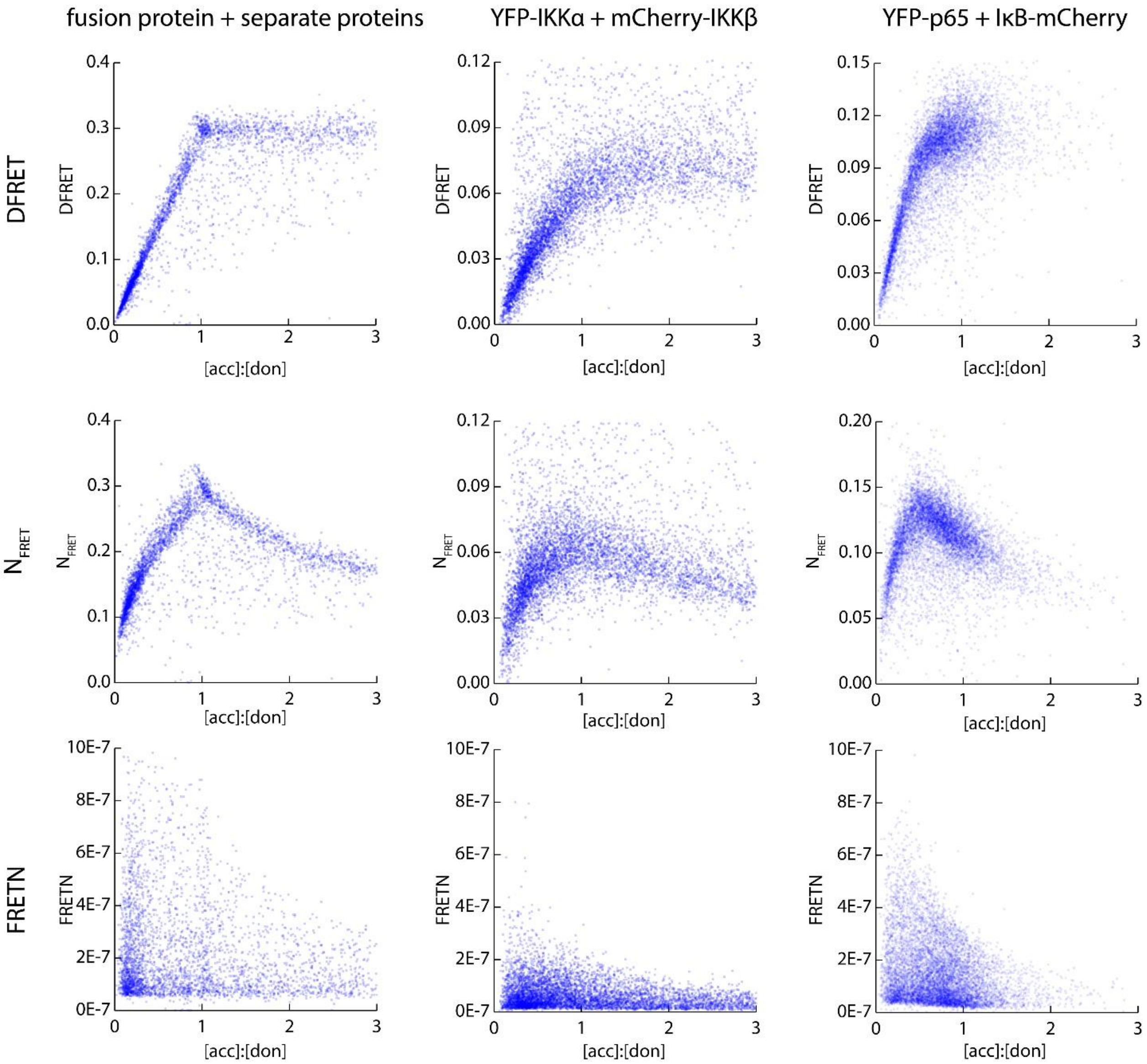

Supplementary Figure 5.

Results of flow cytometry based FRET methods as depicted in different normalization methods. Results in DFRET (top), NFRET (middle row) and FRETN (bottom). Results from mCherry-YFP fusion protein in combination with different amounts of donor or of acceptor alone (left), YFP-IKKα and mCherry-IKKβ (middle) and YFP-p65 and mCherry-IKB (right). Distribution of values at different acceptor to donor ratios. The 3-order of magnitude difference in FRETN values compared to microscopic results (Suppl. Fig 3) originates from the different data bit depth provided by a flow cytometer (10^7 – as compared to 4 x 10^3 of the 12-bit microscopy detector) that is not corrected in FRETN due to the use of the product of donor and acceptor in the denominator during normalization.

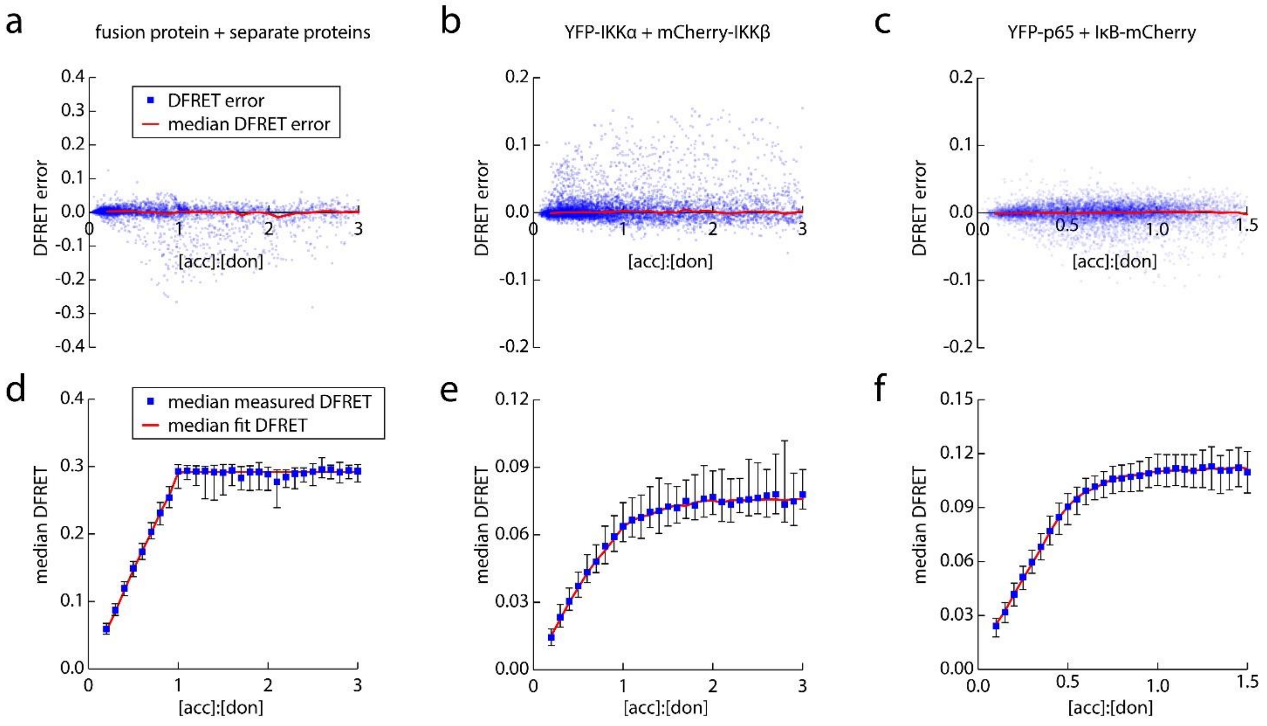

Supplementary Figure 6.

Additional information obtained from the model fit of flow cytometry based DFRET results. Results from mCherry-YFP fusion protein in combination with different amounts of donor or of acceptor alone (left), YFP-IKKα and mCherry-IKKβ (middle) and YFP-p65 and mCherry-IKB (right). (a, b, c) Error of the model fit. Each blue point represents a single measured cell. Median error along the entire population is shown as red line. (d, e, f) Overlap of the median of measured DFRET data and results from the model fit. Blue dots and whiskers represent median and lower and upper quartile of measured data, red line represents median of the results from the model fit.

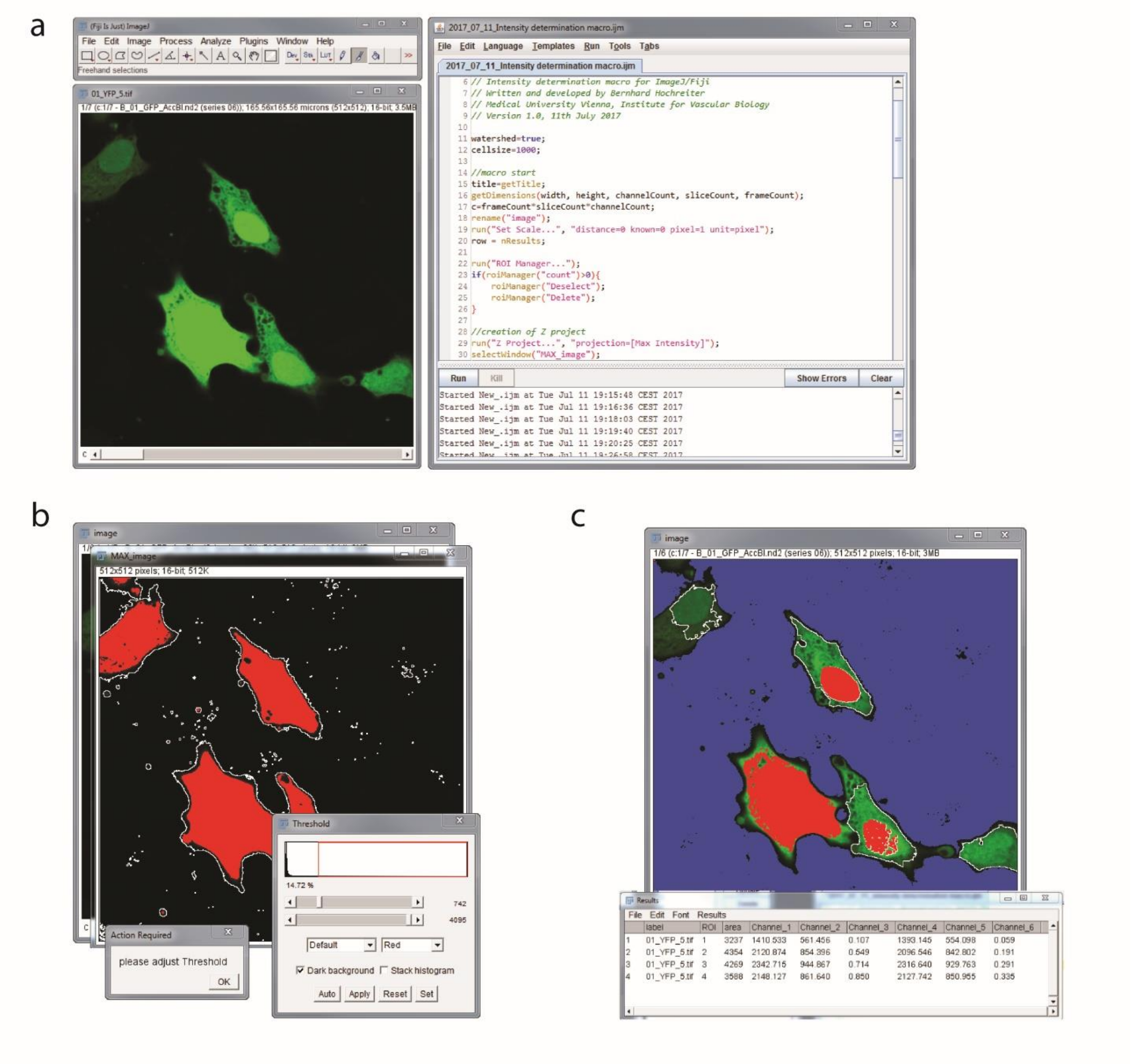

Supplementary Figure 7.

Demonstration of the image evaluation using Fiji and the supplied custom macro. (a) Image and macro are loaded into Fiji by drag & drop onto the window. Image must be in the form of a stack, with single slices containing the different channels. Order of images is irrelevant in this macro as it will only give intensities of objects in the exact order provided. Detected cell size and watershed function can be adjusted on the top of the macro. Cell size gives the minimal pixel size a cell has to cover in order to be accepted as an object for analysis. The watershed function can be turned on (TRUE) or off (FALSE). When turned on, the program will separate single cells by this watershed function. Macro can be started with the run button and will then automatically detect background and remove it and detect cell via a threshold. (b) Macro will stop and prompt to control the applied threshold. Threshold can be adjusted manually, but it is highly advised to keep one predefined threshold mode in order to obtain comparable and reproducible results. (c) Macro will finish evaluation and yield a results window containing extracted data. Image will show detected background in blue, overexposed (and excluded from analysis) pixels in red, and detected and measured cells with white outlines.

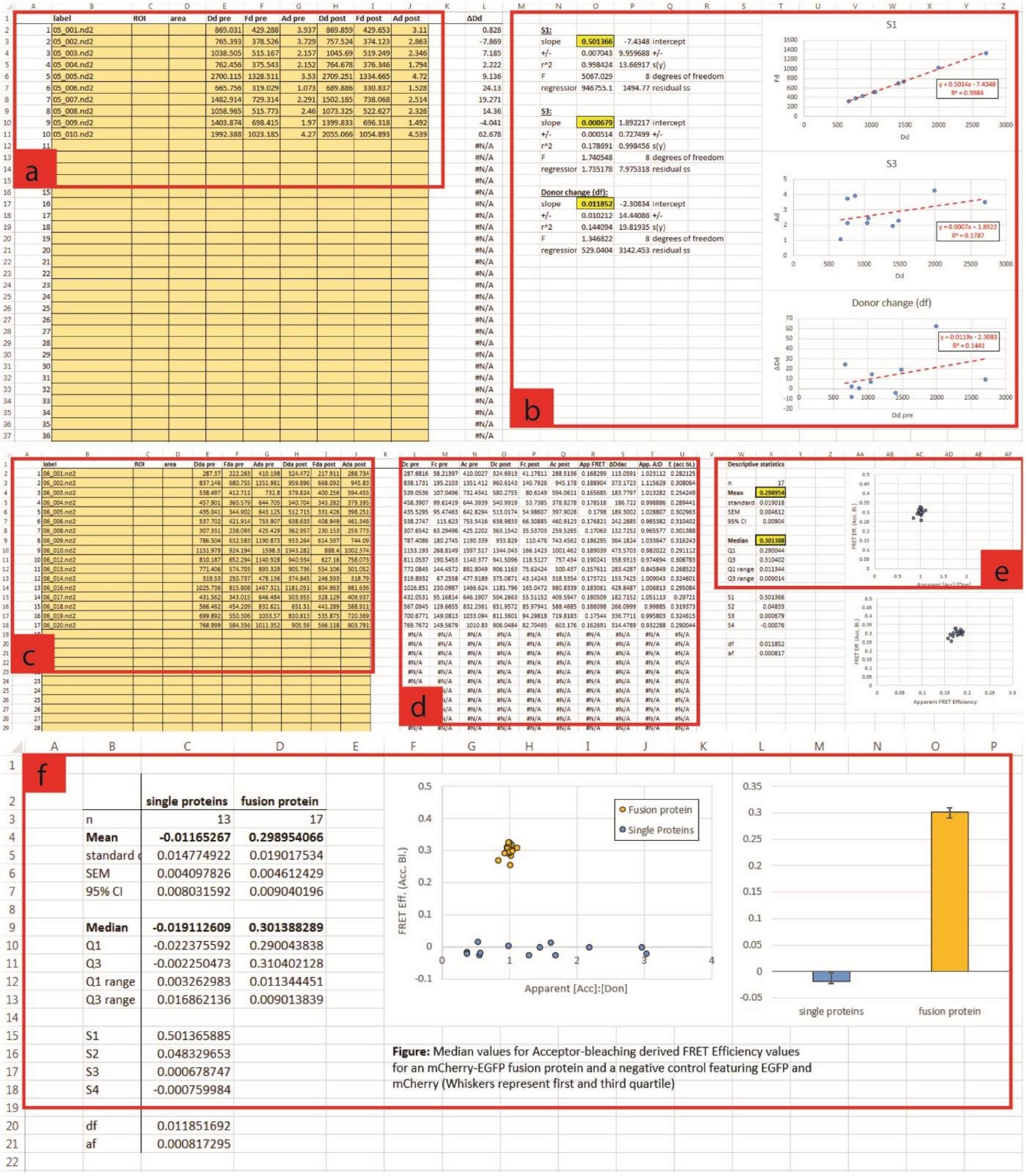

Supplementary Figure 8.

Calculation of FRET Efficiency via acceptor photobleaching, using the provided excel sheet. (a) Raw data values are copied into the respective table in the spreadsheet. donor only, acceptor only, negative control and fusion protein data each have their own spreadsheet. (b) Values for donor change and acceptor photoswitching are automatically calculated. If a FRET signal is provided, sheet will automatically calculate spectral bleed-through factors S1 to S4. (c) From the values of negative control and fusion protein, FRET

efficiency will automatically be calculated (d) and displayed as numerical values, a dot plot (e) and bar chart (f).

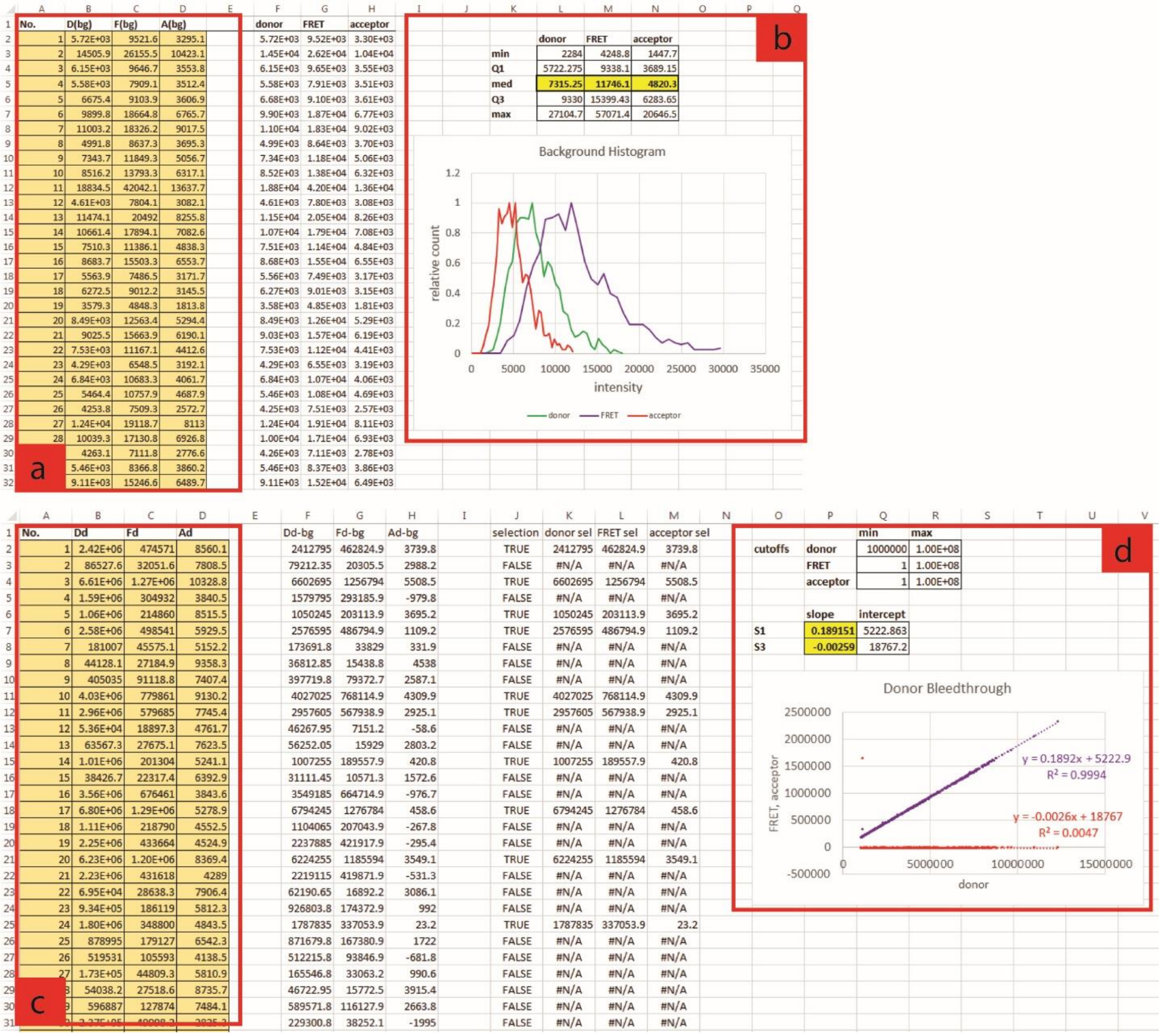

Supplementary Figure 9.

Determination of FRET results using the supplied Excel sheet (part 1). (a) If necessary, values of negative cells are entered. (b) Background values are automatically calculated and removed from all subsequently entered data. (c) Analysis values from samples containing only donor or only acceptor are entered into the respective spreadsheet, and resulting variables S1, S2, S3 and S4 are automatically calculated (d).

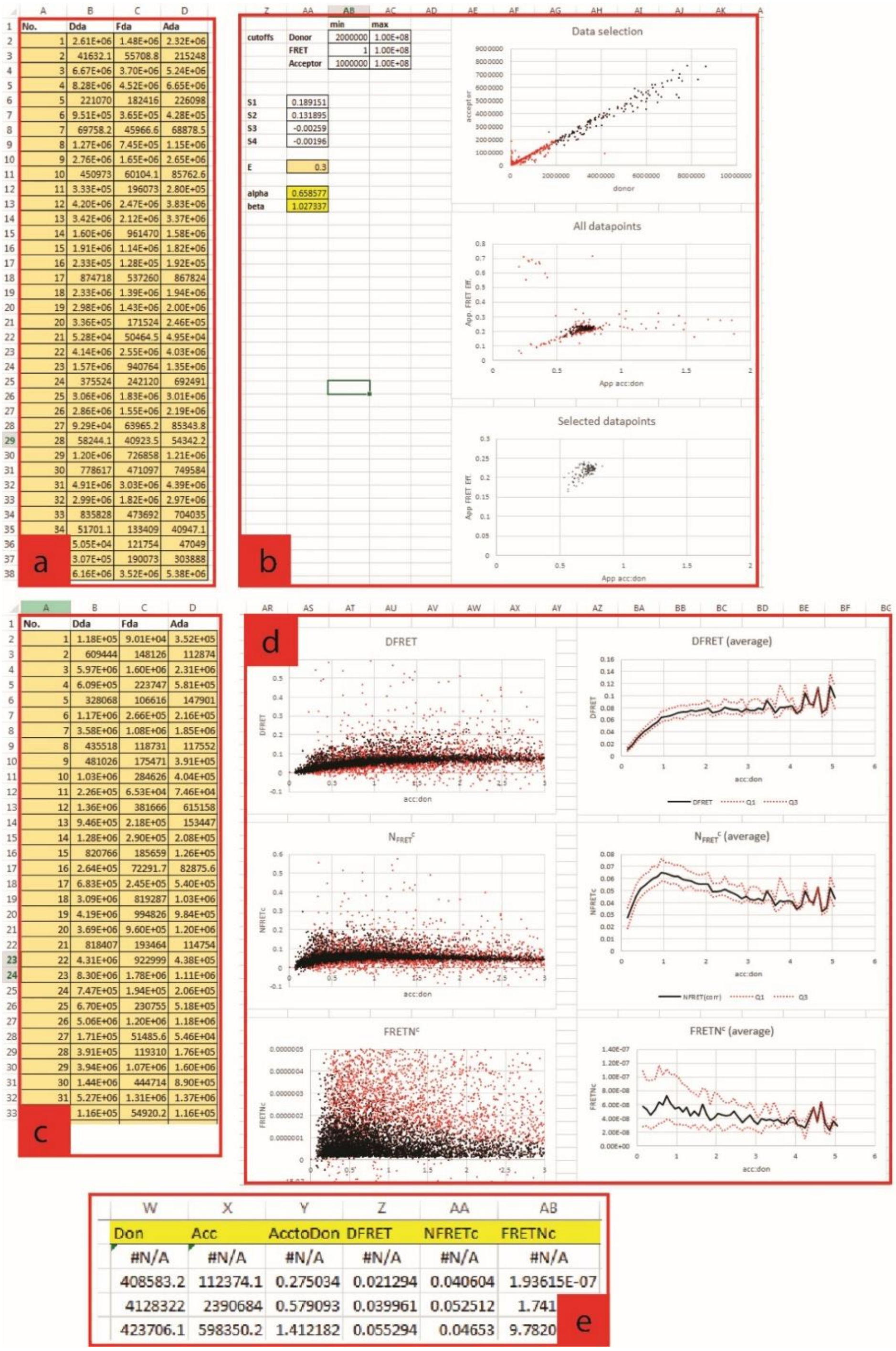

Supplementary Figure 10.

Determination of FRET results using the supplied Excel sheet (part 2). (a) Analysis values from Sample only containing the fusion construct with known FRET Efficiency are entered. (b) Resulting correction factors α and β will be calculated automatically and applied to all subsequent calculations accordingly. (c) Data from interacting sample analysis are entered into the respective sample sheet. Results are automatically depicted as scatterplots (d) and as numerical values (e). Numerical values can be copied into a tab delimited txt file or saved as csv file for subsequent model fitting in R.

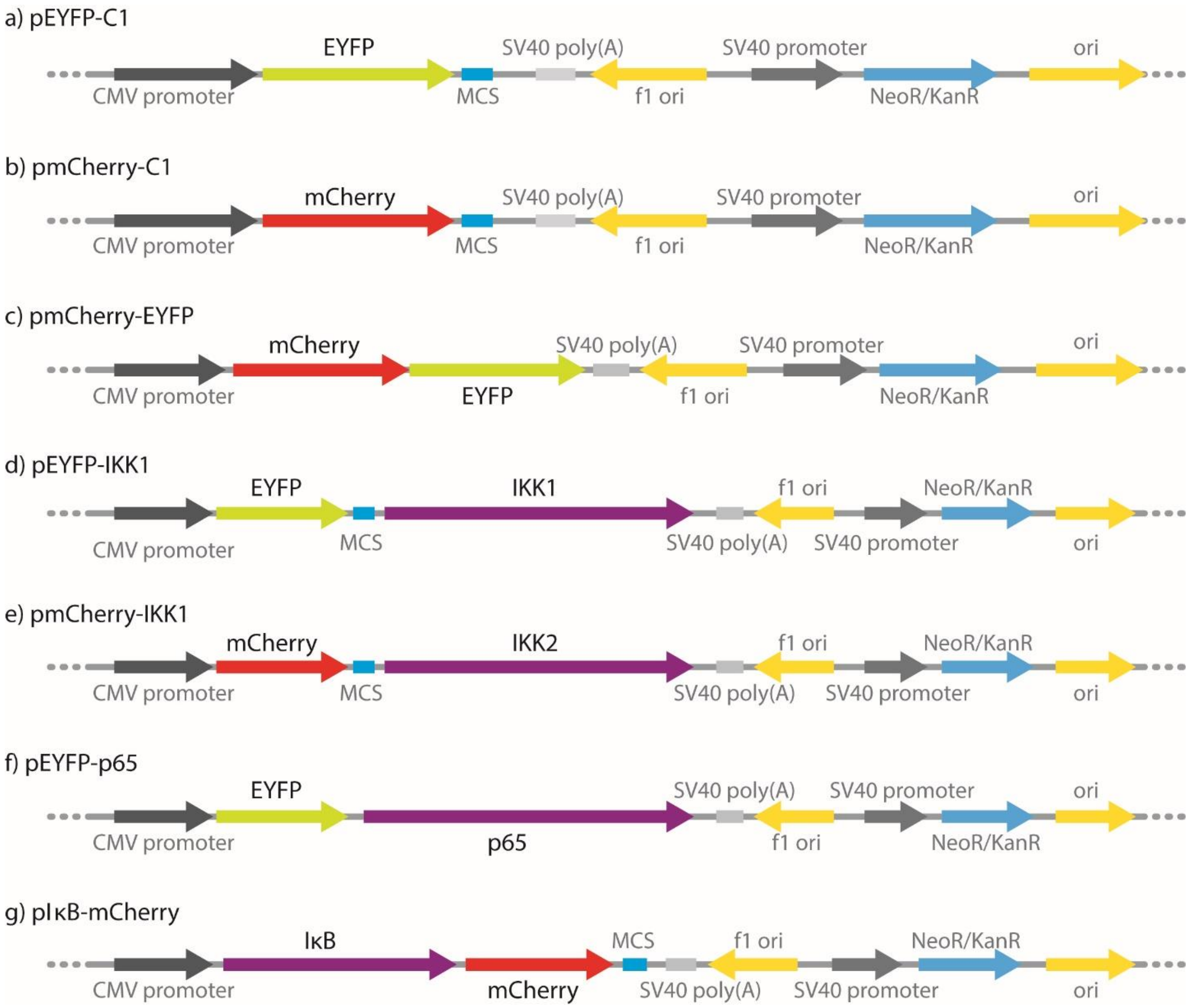

Supplementary Figure 11.

Plasmid maps of used constructs

Supplementary Note 1. Fiji Macro for microscopy evaluation

```
// Intensity determination macro for ImageJ/Fiji
// Written and developed by Bernhard Hochreiter
// Medical University Vienna, Institute for Vascular Biology
// Version 1.0, 11th July 2017

watershed=true;
cellsize=2000;

//macro start
title=getTitle;
getDimensions(width, height, channelCount, sliceCount, frameCount);
c=frameCount*sliceCount*channelCount;
rename("image");
run("Set Scale...", "distance=0 known=0 pixel=1 unit=pixel");
row = nResults;

run("ROI Manager...");
if(roiManager("count")>0){
        roiManager("Deselect");
        roiManager("Delete");
}

//creation of Z project
run("Z Project...", "projection=[Max Intensity]");
selectWindow("MAX_image");

//background detection
setAutoThreshold("Triangle");
run("Create Selection");
roiManager("Add");
roiManager("Select", 0);
roiManager("Rename", "background");
run("Select None");
resetThreshold();

//overexposure detection
run("Duplicate...", "title=overexposure");
setPixel(0,0,4095);
setThreshold(3800, 4095);
run("Convert to Mask");
run("Dilate");
run("Create Selection");
roiManager("Add");
roiManager("Select", 1);
roiManager("Rename", "overexposure");
run("Select None");
close("overexposure");

//detect cells and remove overexposure
selectWindow("MAX_image");
run("Threshold...");
setAutoThreshold("Huang dark");
waitForUser("please adjust Threshold");
run("Convert to Mask");
if(watershed==true){run("Watershed");}
roiManager("Select", 1);
run("Multiply...", "value=0.000");
run("Select None");
run("Analyze Particles...", "size=cellsize-Infinity add");

roiManager("Sort");
roiManager("Select",roiManager("count")-1);
run("Make Inverse");
roiManager("Update");

for(i=0;i<roiManager("count")-2;i++){
```

```
        roiManager("Select", newArray(i,roiManager("count")-1));
        roiManager("AND");
        roiManager("Update");
        roiManager("Deselect");
        roiManager("Select",i);
        roiManager("Rename", i+1);
}

//cell analysis
close("MAX_image");
selectWindow("image");

for(i=0;i<roiManager("count")-2;i++){
        r = row+i;
        roiManager("Select", i);
        getStatistics(area);
        setResult("label", r, title);
        setResult("ROI", r, i+1);
        setResult("area", r, area);
        for(j=1;j<=c;j++){
                setSlice(j);
                roiManager("Select", roiManager("count")-2);
                getStatistics(area, bg);
                roiManager("Select", i);
                roiManager("Set Color", "white");
                getStatistics(area, mean);
                setResult("Channel_"+j, r, mean-bg);
        }
}

roiManager("Select",roiManager("count")-1);
run("Make Inverse");
roiManager("Update");

//image depiction
roiManager("Set Color", "white");
roiManager("Select", roiManager("count")-1);
roiManager("Set Fill Color", "red");
roiManager("Select", roiManager("count")-2);
roiManager("Set Fill Color", "blue");
roiManager("Show All without labels");
roiManager("UseNames", "true");
setSlice(1);
```

Supplementary Note 2. R code for curve fitting of FRET results

```
#R code for curve fitting of FRET results, B. Hochreiter 2018

#please adjust variables:

        separator="\t"                #determines the separator in the raw data file ("\t"=tabulator)

        DFRET=1                       #display DFRET graph? (1=yes, 0=no)
        NFRET=1                       #display NFRET graph? (1=yes, 0=no)
        fitpar="DFRET"                #determine variable used for fitting ("DFRET" or "NFRET")

        Kstart=1e-5                   #enter strat variable for K
        zstart=1                      #enter start variable for z
        Fmaxstart=0.1                 #enter start variable for FRETmax

        lowerAD=0.1                   #enter lower cutoff of acceptor to donor ratio used for fitting
        upperAD=1.5                   #enter upper cutoff of acceptor to donor ratio used for fitting

        printresults=1                #automatically export image into output folder? (1=yes, 0=no)

#DO NOT CHANGE ANYTHING AFTER THIS LINE
#######################################################################################

path<-file.choose()
data1<-read.table(path,header=TRUE,fill=TRUE,sep=separator)
name1<-basename(path)
name<-sub("\\.txt","",name1)
name<-sub("\\.csv","",name)
name2<-paste(sep="",name,"_data.png")
name3<-paste(sep="",name,"_fitting.tif")
name4<-paste(sep="",name,"_FITresults.txt")

#######################################################################################

data1sub=data1[!data1$acctodon>upperAD,]
data1sub=data1sub[!data1sub$acctodon<lowerAD,]

f1<-function(don,acc,K,z,Fmax){(-sqrt((-don*K-acc*z*K-1)^2-
4*don*acc*z*K^2)+don*K+acc*z*K+1)/(2*K*sqrt(don*acc))*Fmax}
f2<-function(don,acc,K,z,Fmax){(-sqrt((-don*K-acc*z*K-1)^2-
4*don*acc*z*K^2)+don*K+acc*z*K+1)/(2*K*don)*Fmax}

if(fitpar=="DFRET"){
        fit<-
nls(DFRET~f2(don,acc,K,z,Fmax),algorithm="port",data=data1sub,start=list(K=Kstart,z=zstart,Fmax=Fmaxstart)
, lower=list(K=1e-20,z=0.01,Fmax=0.01), upper=list(K=1e20,z=10,Fmax=1),control=list(maxiter = 500000,
minFactor=1/2000, warnOnly=T))
        start=coef(fit)

        fit<-
nls(DFRET~f2(don,acc,K,z,Fmax),algorithm="port",data=data1sub,start=list(K=start[1],z=start[2],Fmax=start[
3]), lower=list(K=1e-20,z=0.01,Fmax=0.01),control=list(maxiter = 50000, minFactor=1/2000, warnOnly=T))
}else if(fitpar=="NFRET"){
        fit<-
nls(NFRET~f1(don,acc,K,z,Fmax),algorithm="port",data=data1sub,start=list(K=Kstart,z=zstart,Fmax=Fmaxstart)
, lower=list(K=1e-20,z=0.1,Fmax=0.01), upper=list(K=1e20,z=3,Fmax=1),control=list(maxiter = 500000,
minFactor=1/2000, warnOnly=T))
        start=coef(fit)

        fit<-
nls(NFRET~f1(don,acc,K,z,Fmax),algorithm="port",data=data1sub,start=list(K=start[1],z=start[2],Fmax=start[
3]),lower=list(K=1e-20,z=0.1,Fmax=0.01), upper=list(K=1e20,z=3,Fmax=1),control=list(maxiter = 50000,
minFactor=1/2000, warnOnly=T))
}

fitv<-coef(fit)
NFRETfit=f1(data1sub$don,data1sub$acc,fitv[1],fitv[2],fitv[3])
```

```
DFRETfit=f2(data1sub$don,data1sub$acc,fitv[1],fitv[2],fitv[3])
DFRETres=data1sub$DFRET-DFRETfit
NFRETres=data1sub$NFRET-NFRETfit

yupb=2*fitv[3]
if(DFRET+NFRET==1){
dev.new(width=6,height=3)
old.par=par(mfrow=c(1, 2))
}

if(DFRET+NFRET==2){
dev.new(width=6,height=6)
old.par=par(mfrow=c(2, 2))
}


if(DFRET==1){
        plot(data1sub$acctodon,data1sub$DFRET,ylim=c(0,yupb),xlim=c(0,3),xlab="[acc]:[don]",ylab="DFRET",c
ol=rgb(0,0,0,alpha=0.5),pch=20, main="original data",cex.lab=1.5, cex.axis=1.5, cex.main=1.5, cex.sub=1.5)
        points(data1sub$acctodon,DFRETfit,col=rgb(1,0,0,alpha=0.4),pch=20)

        plot(data1sub$acctodon,DFRETres,xlim=c(0,3),ylim=c(-
yupb,yupb),col=rgb(0,0,1),xlab="[acc]:[don]",ylab="DFRET residuals",pch=15,main="residuals",cex.lab=1.5,
cex.axis=1.5, cex.main=1.5, cex.sub=1.5)
        lines(c(0,3),c(0,0),col="red")

}


if(NFRET==1){
        if(DFRET==0){ main1="original data";main2="residuals"}else{main1="";main2=""}

        plot(data1sub$acctodon,data1sub$NFRET,ylim=c(0,yupb),xlim=c(0,3),xlab="[acc]:[don]",ylab="NFRET",c
ol=rgb(0,0,0,alpha=0.5),pch=20, main=main1,cex.lab=1.5, cex.axis=1.5, cex.main=1.5, cex.sub=1.5)
        points(data1sub$acctodon,NFRETfit,col=rgb(1,0,0,alpha=0.4),pch=20)

        plot(data1sub$acctodon,NFRETres,xlim=c(0,3),ylim=c(-
yupb,yupb),col=rgb(0,0,1),xlab="[acc]:[don]",ylab="NFRET residuals",pch=15,main=main2,cex.lab=1.5,
cex.axis=1.5, cex.main=1.5, cex.sub=1.5)
        lines(c(0,3),c(0,0),col="red")

}
if(DFRET+NFRET==1){if(printresults==1){dev.print(tif,file=name3,unit="px",width=600,height=300)}}
if(DFRET+NFRET==2){if(printresults==1){dev.print(tif,file=name3,unit="px",width=600,height=600)}}


par(old.par)
summary(fit)

sink(name4)
cat(name)
summary(fit)
sink()
```
